# Diversity, Prevalence, and Longitudinal Occurrence of Type II Toxin-Antitoxin Systems of *Pseudomonas aeruginosa* Infecting Cystic Fibrosis Lungs

**DOI:** 10.3389/fmicb.2017.01180

**Published:** 2017-06-23

**Authors:** Sandra B. Andersen, Melanie Ghoul, Ashleigh S. Griffin, Bent Petersen, Helle K. Johansen, Søren Molin

**Affiliations:** ^1^Department of Zoology, University of OxfordOxford, United Kingdom; ^2^The Novo Nordisk Foundation Center for Biosustainability, Technical University of DenmarkLyngby, Denmark; ^3^Department of Bio and Health Informatics, Technical University of DenmarkLyngby, Denmark; ^4^Department of Clinical Microbiology, RigshospitaletCopenhagen, Denmark

**Keywords:** toxin-antitoxin system, *Pseudomonas aeruginosa*, cystic fibrosis, infection, longitudinal studies, chronic infection, genomic islands, integrative and conjugative elements

## Abstract

Type II toxin-antitoxin (TA) systems are most commonly composed of two genes encoding a stable toxin, which harms the cell, and an unstable antitoxin that can inactivate it. TA systems were initially characterized as selfish elements, but have recently gained attention for regulating general stress responses responsible for pathogen virulence, formation of drug-tolerant persister cells and biofilms—all implicated in causing recalcitrant chronic infections. We use a bioinformatics approach to explore the distribution and evolution of type II TA loci of the opportunistic pathogen, *Pseudomonas aeruginosa*, across longitudinally sampled isolates from cystic fibrosis lungs. We identify their location in the genome, mutations, and gain/loss during infection to elucidate their function(s) in stabilizing selfish elements and pathogenesis. We found (1) 26 distinct TA systems, where all isolates harbor four in their core genome and a variable number of the remaining 22 on genomic islands; (2) limited mutations in core genome TA loci, suggesting they are not under negative selection; (3) no evidence for horizontal transmission of elements with TA systems between clone types within patients, despite their ability to mobilize; (4) no gain and limited loss of TA-bearing genomic islands, and of those elements partially lost, the remnant regions carry the TA systems supporting their role in genomic stabilization; (5) no significant correlation between frequency of TA systems and strain ability to establish as chronic infection, but those with a particular TA, are more successful in establishing a chronic infection.

## Introduction

Bacterial genomes contain flexible regions that vary in the extent to which they serve their own selfish interests to ensure transmission or confer a selective advantage to the cell (Doolittle, 2005; Lawrence and Hendrickson, 2005). Type II toxin-antitoxin (TA, hereafter used only to refer to type II) systems are one such example. They are comprised of a typically bicistronic operon encoding two proteins: a stable toxin that harms the host cell, and its cognate unstable antitoxin that inactivates the toxin by binding to it (Gerdes et al., 2005). TA systems are near ubiquitous and highly diverse in bacterial species (Pandey and Gerdes, 2005; Shao et al., 2011). They were first discovered and described for their ability to stabilize plasmids by selectively killing plasmid-free daughter cells (Gerdes et al., 1986). This mechanism is termed post-segregational killing and is a result of the differential stability of the toxin and antitoxin: if the plasmid is lost, the antitoxin is no longer produced, and subsequently the toxin can interact with its cellular target (Gerdes et al., 1986; Yarmolinsky, 1995). In such cases, TA systems act as selfish elements that ensure inheritance of self and neighboring genes.

Subsequently, TA systems have been identified in abundance on bacterial chromosomes where they may serve different functions (Tsilibaris et al., 2007; Saavedra De Bast et al., 2008). Compatible with the plasmid stabilization hypothesis, TA systems have been found to stabilize chromosomal mobile elements and genomic islands on which they reside, such as integrative conjugative elements (ICEs) and super-integron cassettes (Pandey and Gerdes, 2005; Christensen-Dalsgaard and Gerdes, 2006; Mazel, 2006; Qiu et al., 2006; Szekeres et al., 2007; Tsilibaris et al., 2007; Wozniak and Waldor, 2009). However, this is not the case for TA systems on other chromosomal regions (Wilbaux et al., 2007). For example, TA systems residing in the core genome are receiving growing interest for their role in the development of recalcitrant chronic infections (Shah et al., 2006; Lewis, 2008; Singh et al., 2009; Wang and Wood, 2011; Lobato-Marquez et al., 2016).

TA systems have been implicated as particularly important in the bacterial general stress response (Buts et al., 2005; Gerdes et al., 2005; Ramage et al., 2009). However, Tsilibaris et al. (2007) show that the TA systems of *Escherichia coli* did not contribute to the bacterial stress response or give cells a selective advantage when in stressful conditions. TA-induced stress-regulation is increasingly studied in pathogens as a mechanism for enhancing virulence (Ramage et al., 2009; Norton and Mulvey, 2012; Ren et al., 2012, 2014; De La Cruz et al., 2013); inducing a reversible bacteriostatic persister state in response to antibiotic treatment or host immunity; rendering cells multidrug tolerant or resistant to macrophages (Christensen et al., 2001, 2003; Lewis, 2008; Wang and Wood, 2011; Yamaguchi et al., 2011; Fasani and Savageau, 2013; Helaine et al., 2014; Helaine and Kugelberg, 2014; Maisonneuve and Gerdes, 2014; Page and Peti, 2016; Vogwill et al., 2016); and inducing biofilm formation (Gonzalez Barrios et al., 2006; Kim et al., 2009; Wang and Wood, 2011). It has been shown that pathogenic strains of chronic infections carry more TA systems than related non-pathogenic species (Ramage et al., 2009; Georgiades and Raoult, 2011; Sala et al., 2014; Lobato-Marquez et al., 2015), however, Pandey and Gerdes (2005) found that obligate intracellular bacteria often lack TAs. Further, in some systems, the cumulative number of TA systems harbored is positively correlated with the ability to induce the persister state (Maisonneuve et al., 2011; Fasani and Savageau, 2013; Vogwill et al., 2016).

Despite extensive research exploring the numerous biological functions of chromosomal TA systems, their role as selfish or stabilization elements and in pathogenic stress physiology is the subject of on-going study. Here we present the largest intraspecies study of type II TA systems, to our knowledge, of the opportunistic pathogen *Pseudomonas aeruginosa*. Some TA systems have previously been identified from *P. aeruginosa* computationally, and found chromosomally and on mobile elements, either integrated as prophages and ICEs or on plasmids (Webb et al., 2004; Pandey and Gerdes, 2005; Ramirez-Diaz et al., 2011; Ou et al., 2012; Bonnin et al., 2013; Li et al., 2016a). Two systems, HicBA and HigAB, were recently confirmed experimentally (Li et al., 2016b; Wood and Wood, 2016). We use a collection of cystic fibrosis (CF) lung isolates that are longitudinally sampled from Danish patients to follow the evolution of TA loci in lineages during infection, and compare them to environmental isolates, in an attempt to determine their location and function in the accessory genome and subsequently infer whether they play a role in adaptation to chronic infection conditions.

With a bioinformatics approach we explore the prevalence of type II TA systems across *P. aeruginosa* isolates. The five main aims are to: (1) Identify the diversity of TA systems and whether they reside in the core genome or on mobile and/or flexible genomic islands (GIs); (2) examine whether TA loci acquire mutations during infection; (3) track changes in the abundance of systems as strains reside in the lung; (4) screen for whether there is evidence of horizontal transmission of TA systems between coexisting clone types; (5) test whether the number and type of TA systems correlate with how successful strains are at establishing chronic infections, potentially because of their ability to form persisters recalcitrant to antibiotic treatment.

## Materials and methods

### Isolate collection

We used *P. aeruginosa* whole genome sequencing of isolates longitudinally sampled from 33 young Danish CF patients, as described previously (Marvig et al., 2013, 2015b). Patients were either infected with one clone type that was sampled repeatedly over many years, harbored co-occurring clone types, or were first colonized by one clone type that was replaced by another with no detected co-occurrence (Marvig et al., 2015a,b). The collection contained sequence data from 441 isolates of 50 clone types. The genomes of *P. aeruginosa* from the young patients were *de novo* assembled, as described previously (Marvig et al., 2015a) and are available at the European Nucleotide Archive, accession number PRJEB5438 (www.ebi.ac.uk/ena). The genomes were not assembled to one chromosome but were comprised of multiple contigs (for clinical isolates on average 136 ± 274 *SD*, Table S1), which may result in not all TA systems being identified. For each contig open reading frames (ORFs) were predicted with the program Prodigal (Prodigal.ornl.gov; Hyatt et al., 2010). In addition, we included two transmissible clone types, DK1 (41 isolates) and DK2 (44 isolates), which were transmitted between patients from 1973 up to the 1990's as previously described (Jelsbak et al., 2007; Yang et al., 2011; Marvig et al., 2013; Markussen et al., 2014). These were primarily from older patients with one or a few isolates from each. In younger patients, following measures to separate patients in the clinic, the majority of Danish CF infections are caused by environmental strains. To test whether TA systems of the *P. aeruginosa* that establish CF infection differ from those that do not, we included 12 environmental isolates collected from non-infectious sources described in Hilker et al. (2015; sequences available at ENA accession no. PRJEB4961 and *de novo* assemblies kindly provided by J. Klockgether) and Ghoul et al. (2015; sequences and *de novo* assemblies available at http://pubmlst.org/paeruginosa/; accession no. WTCHG-44355_209 to 213). The clone types of environmental isolates were determined following Marvig et al. (2015b); three were found to overlap with clone types found in clinical isolates while nine were distinct new types.

### Identification of putative TA systems and sequence similarity within gene families

*De novo* genome assemblies of the first isolate of each clone type from each patient were analyzed to find the number and family of putative TA loci, for a total of 69 first isolates of 52 clone types from 35 patients, and 12 environmental isolates of 12 different clone types. Putative type II toxin-antitoxin systems were identified with the program TAfinder (http://202.120.12.133/TAfinder/index.php; Shao et al., 2011). The cut-off was set at a score of an average shared identity of the toxin and the antitoxin at 80% with previously computationally and experimentally identified TA systems. Systems classified by TAfinder were subjected to BLASTP analysis to determine conserved domains, and a BLASTP analysis excluding Pseudomonas hits to see if they were found, as a pair, in other species. TAfinder failed to annotate as open reading frames one toxin (HigB) and one antitoxin (ParD) that had previously been identified. The antitoxin HigA (PA4674) and the toxin ParE (PA0729) were collected from the ORF files as described below. The coding sequences of the unannotated toxin and antitoxin were identified by a BLASTN search against a local database of all the clinical isolates, and these were translated to protein sequences with EMBOSS Transeq (http://www.ebi.ac.uk/Tools/st/emboss_transeq/) for the first isolate from each clone type. A third previously identified pair, HicBA, was identified by BLAST search of the toxin sequence against the *de novo* assemblies. The diversity within a given toxin and antitoxin was calculated in Clustal Omega (http://www.ebi.ac.uk/Tools/msa/clustalo/), and unique protein sequences were identified with GenomeTools (http://genometools.org/) and compared to known sequences with BLASTP. The unique sequences were aligned with the reference sequence from TAfinder with Clustal Omega. Protein sequences without a 100% identity match were submitted to GenBank. We refer to all of these putative TA systems as TA systems, but note that for the majority their function still needs to be confirmed experimentally.

### Location and neighboring genes of TA systems

The TA systems not found in all isolates were considered to be part of the accessory genome, on GIs and potentially mobile elements. The location of TA systems in the *P. aeruginosa* accessory genome was determined by BLASTP analysis restricted to the PAO1 reference genome. We attempted to identify where GIs were inserted into the core-genome: If a TA pair was found on a 100 kb contig, the predicted ORFs of the entire contig were subjected to BLASTP analysis against the reference PAO1 genome. A potential GI insertion site was determined when genes with high shared identity to genes in PAO1 (>90%) were found next to genes with lower shared identity (>40%). The insertion site was confirmed by identifying an upstream gene with high similarity to a gene in PAO1, neighboring the suspected insertion site. The inferred insertion sites of GIs were compared to those identified by Mathee et al. (2008).

### Identification of TA loci mutations during infection

To identify mutations in TA loci acquired during infection we compared isolates of the same clone type sampled longitudinally from patients. We aligned *de novo* assembled contigs of the region of interest using BLAST align or mapped raw reads to representative sequences of the clone type. The reads were mapped to reference sequences with the Burrows-Wheeler alignment tool (http://bio-bwa.sourceforge.net, version 07.4) and alignments generated with the paired-end reads or single-end reads setting, respectively. The alignments were filtered to remove unmapped reads, sorted and indexed, and assigned to a read group using Picard Tools (http://picard.sourceforge.net, version 1.91). Differences between isolates of the same clone type were identified using Samtools (http://samtools.sourceforge.net, version 01.19) and mutations manually checked using IGV (http://www.broadinstitute.org/igv, version 2.3.61).

### Rates of gain and loss of TA-bearing GIs during infection and on an ecological time scale

Deletions and insertions of >50 bp in GIs were identified in the sequence alignments described above. To identify gain of TA systems during infection we used the first 5–8 amino acids of a toxin or antitoxin gene, provided by TAfinder, as a search motive to collect the genes from all isolates from the young patients from the files with predicted ORFs. The use of these short motives allowed for discovery of TAs already identified in other isolates that potentially had been vertically transmitted between clone types within patients, and the identity of positive hits was verified by BLASTP search.

Nine clone types found in multiple patients has been shown not to be transmitted among patients, but independently acquired by different patients from the environment (Marvig et al., 2015b; Table S1). To estimate the timescale of gain and loss of GIs with TAs in the environment we used the variation in presence of GIs of these isolates. The number of SNPs between isolates (Marvig et al., 2015b) was used as a measure of time since the last common ancestor, given an estimated average rate of 2.5 SNPs/year (Yang et al., 2010). The three environmental isolates of the same clone types as clinical isolates were not included in this analysis because the sequence coverage was lower, and the SNPs identified therefore not reliable (but see Table S2 for comparisons).

### Correlation between TA systems and infection success

All clone types from the young patients were classified based on their success in establishing an infection; where “low” was used for clone types sampled from a patient once, or multiple times over less than a year (*n* = 29), “medium” for clone types sampled multiple times infecting for longer than a year but either co-occurring with other clone types or being replaced (*n* = 16) and “high” for clone types consistently sampled with a frequency above 80% of sampled isolates from a patient infecting for more than a year (*n* = 24). These were compared with the 12 environmental samples. We used 1-Way ANOVA to test if the number of TA systems were significantly different between categories and a χ^2^-test to analyse if the presence of GIs varied significantly between categories.

## Results

### Diversity of TA systems across isolates determined by the presence of TAs residing on GIs

All isolates harbored TA systems, and we found extensive diversity across the studied isolates. TAfinder identified 32 distinct TA systems, by screening all first sampled isolates of clone types from all patients (69 isolates of 52 clone types from 35 patients) and 12 environmental isolates. Of these, 8 antitoxins, 18 toxins, and 7 pairs were verified by BLASTP analysis, as they produced a specific hit to conserved domains of toxin or antitoxins. Sixteen T and As had an unspecific hit to conserved domains of toxin or antitoxins [*E* < 0.01, Table S3 and Supporting Online Material (Data Sheet 1)]. For the putative TAs without a clear match we did a BLASTP analyses with an Entrez Query specified as toxin or antitoxin, and all except two shared >27% identity with putative TAs from various species [Table S3 and Supporting Online Material (Data Sheet 1)].

**Figure 1:**
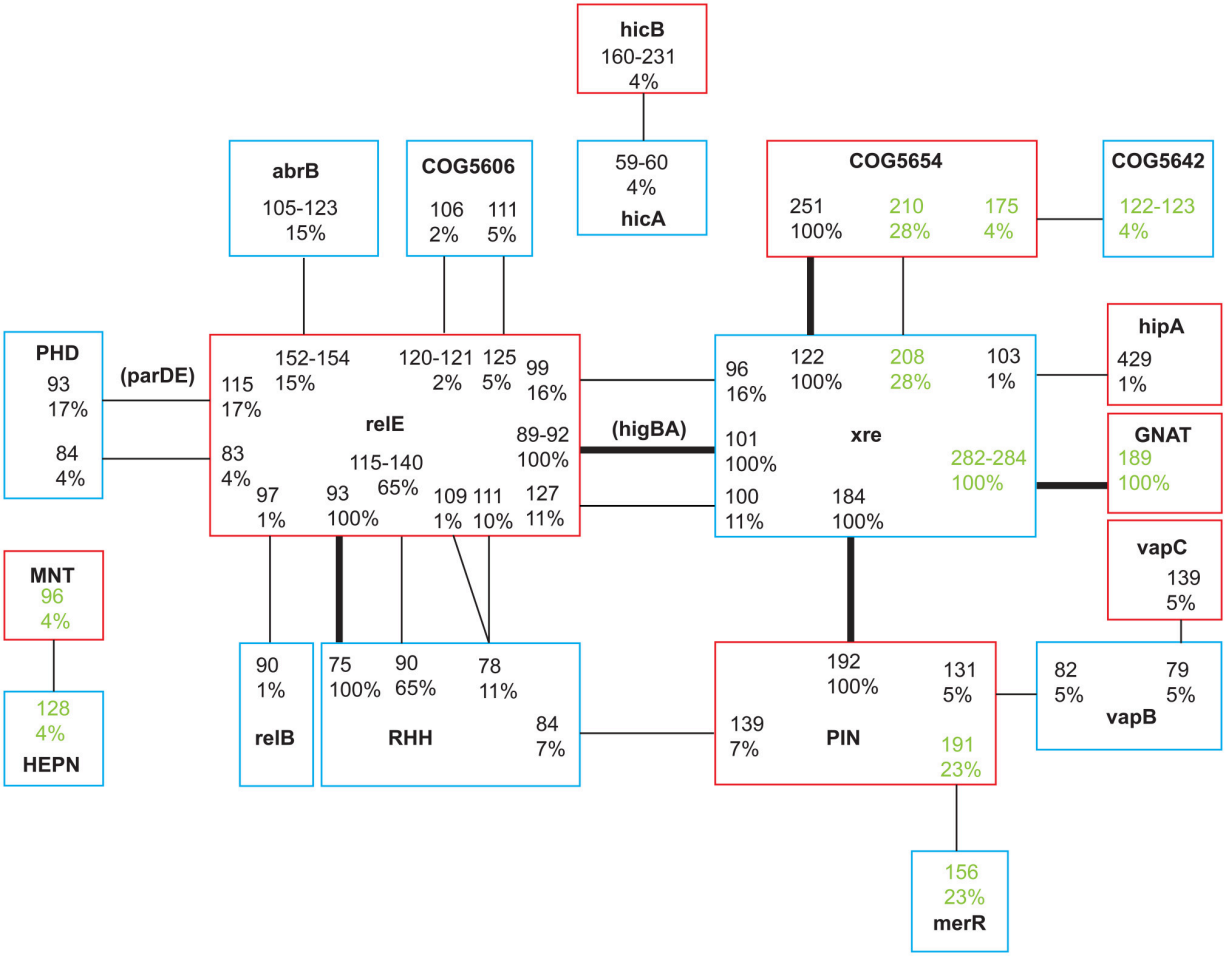
Network of toxin and antitoxin associations categorized by family or domain type. Toxins are marked with red and antitoxins blue. Each TA pair is denoted by a number giving the length of the protein in amino acids and the prevalence in the study population in %. Lines mark the connection between a toxin and its antitoxin. Bold lines mark TA systems found in the core genome of all isolates. Two TA pairs were named by Pandey and Gerdes (2005), these names are denoted in parentheses. Pairs identified by TAfinder but not verified by BLASTP analysis are highlighted in green. Seven unique TA pairs were only found in one environmental isolate (Tables S1, S3) and are not included in the figure.

We classify the TA systems into four groups based on whether the T and A in a pair are both verified, only one partner is verified or both unverified: in category 1 both the toxin and antitoxin were verified by a specific hit by BLASTP analysis (seven pairs), or experimentally confirmed (two pairs); in category 2 either the toxin or antitoxin has a specific hit but the partner does not (10 pairs); in category 3 one partner has an unspecific hit and the partner does not (seven pairs); and in category 4 neither were verified (six pairs). These latter six pairs, however, all had conserved domains that Makarova et al. (2009) identified as strong candidates for TA systems. Makarova et al. (2009) suggest that neighboring genes to known T or As present strong candidates for novel components, and in the subsequent analyses, we include all pairs from TAfinder where one or both components were verified by unrestricted BLASTP analysis. We leave the category 4 pairs out of the subsequent analyses, although we note that these may be functional TAs given their sequence similarity to putative systems from other species. For that reason, they are listed in Tables S1, S3 and Figure 1, and when mentioned in the text referred to as unverified systems. As further support for the identified pairs being TA systems, all were also found, as pairs, in a diverse range of other species (Table S3). Each isolate harbored between 4 and 11 systems of categories 1–3 (mean 5.89 systems ± 1.64 *SD*, Table S1). Four TA systems were part of the core genome: PA1029/PA1030 (Xre/COG5654, category 3), PA1878/PA1879 (Xre/PIN, category 3), PA0124/PA0125 (RHH/RelE, category 2), and PA4674/ unannotated (HigAB, category 1; Tables 1, 2; found in all environmental and first sampled clinical isolates, except one that was missing the HigAB pair). For the Xre/COG5654 and the Xre/PIN systems the toxins were not classified as such by BLASTP analysis, but the PIN toxin shared 68% identity with previously identified toxins in the restricted search. The RHH/RelE and HigAB pairs had been identified previously (Pandey and Gerdes, 2005), and the latter confirmed experimentally (Wood and Wood, 2016). All of these core genome pairs were also found in the *P. aeruginosa* reference genomes PA1, PA7, UCBPP-PA14, and LESB58. The other 22 TA systems varied in prevalence from 1% (found in one clone type in one isolate) to 65% (1–2 copies in 48 first sampled isolates of 34 CF clone types and 4 environmental isolates). Our analyses suggest that these are located on GIs with specific insertion sites (Table S3). Therefore, variation in TA systems across isolates was caused by those that reside in the accessory genome, on potentially mobile elements.

**Table 1:** Overview of TA diversity.

**Antitoxin**	**Toxin**	**Size A/T**	**Prevalence %**	**Location**	**A aa % ID**	**T aa % ID**	**Category**
*RHH*	*relE*	75/93	100	CG	98.67–100	94.62–100	Cat2
*xre*	*COG5654*	122/251	100	CG	99.18–100	96.84–100	Cat3
*xre*	*PIN*	184/192	100	CG	95.11–100	96.88–100	Cat3
*xre (higA)*	*relE (higB)*	101/89-92	99	CG	95.05–100	95.65–100	Cat1
*RHH*	*relE*	90/115-140	65	GI	93.33–100	85.34–100	Cat1
*PHD (parD)*	*relE (parE)*	83/115	17	GI	100	97.39–100	Cat1
*xre*	*relE*	96/99	16	GI	100	100	Cat2
*abrB*	*relE*	105-123/152-154	15	GI	71.43–100	79.05–100	Cat1
*xre*	*relE*	100/126-127	11	GI	91–100	86.61–100	Cat2
*RHH*	*relE*	78/111	10	GI	97.30–100	97.2–100	Cat2
*RHH*	*PIN*	84/139	7^*^	GI	97.62–100	42.88–100	Cat2
*COG5606*	*relE*	111/125	5	GI	99.1–100	100	Cat3
*vapB*	*PIN*	83/131	5	GI	100	100	Cat1
*vapB*	*vapC*	79/136	5	GI	93.67–100	94.85–100	Cat3
*hicB*	*hicA*	160-231/59-60	4^**^	GI	62.71–93.22	66.88	Cat1
*PHD*	*relE*	83/84	4	GI	100	100	Cat1
*COG5606*	*relE*	106/120-121	2	GI	97.17	98.33	Cat3
*abrB*	*PIN*	87/129	1	GI			Cat1
*COG5606*	*relE*	112/120	1	GI			Cat3
*hicB*	*hicA*	121/91	1	GI			Cat2
*PHD*	*PIN*	77/128	1	GI			Cat1
*relB*	*relE*	90/97	1	GI			Cat2
*RHH*	*PIN*	73/126	1	GI			Cat2
*RHH*	*relE*	78/109	1	GI			Cat2
*xre*	*hipA*	103/429	1	GI			Cat2
*xre*	*relE*	100/117	1	GI			Cat3

*The first two columns list the gene/domain classes for the antitoxin and toxin pair, followed by the protein length of each in amino acids. Variation in protein length within a class is denoted by a “–.” For each TA pair its prevalence in % of isolates is stated, followed by its location in either the core genome (CG) or on a genomic island (GI), and for each toxin and antitoxin the shared amino acid identity within a group is noted. The pairs are classified as category 1–4 dependent on the presence of conserved T and A domains (category 4 are pairs not included in this table). The toxin and antitoxin classes that have more than two representatives are colored in gray tones*.

* *One toxin appears to be missing antitoxin.

** **Two out of four toxins appear to be missing antitoxins. This table is extended in Table S3 with additional information on TA pairs.

**Table 2:** Mutations in TA systems.

**Event**	**TA loci**	**Loc**.	**Mut**.	**Clone type**	**Patient**	**Isolate**	**Est. del**.	**Infection age**	**Comment**
Insertion in antitoxin	*xre/COG5654*	CG	2C	DK23	P22M4	177		0 years	Isolate sampled as one-off, toxin likely functional
Insertion in toxin	*xre/COG5654*	CG		DK21	P22M4	All			40 kb insertion with phage-like genes
Ns snp in toxin	*xre/relE (higAB)*	CG	C61T	DK01	P28F1	2009		36 years	
Deletion of TA pair	*xre/relE (higAB)*	CG		DK28	P96F4	202	1.6 kb	0 years	Isolate sampled as one-off
Deletion of TA pair	*xre/PIN*	CG		DK13	P05M4	117	>40 kb	2.4 years	
Stop codon in antitoxin	*xre/PIN*	CG	T291A		Env.	MG7			Toxin likely functional
Stop codon in antitoxin	*xre/PIN*	CG	T291A	DK49	P72F4	341		0 years	Isolate sampled as one-off, toxin likely functional
Ns snp in antitoxin	*xre/GNAT*	CG	A439G	DK36	P77F4	400		2.73 years	
Partial loss of GI, TA lost	*COG5642/COG5654*	GI		DK26	P92F3	268, 273–275	105.5 kb	0.35 year	
Partial loss of GI, TA remains	*PHD/relE (parDE)*	GI		DK24	P22M4	183	43 kb	0.17 year	
Partial loss of GI, TA remains	*abrB/relE &* merR/PIN	GI		DK36	P77F4	403	53.4 kb	2.73 years	
Complete loss of GI	*RHH/relE 90/115-140*	GI		DK44	P70F4	334	81 kb	1.06 years	
Complete loss of GI	*RHH/relE 90/115-140*	GI		DK02	P06M2	2002	90.6 kb	29 years	Hypermutator
Complete loss of GI	*RHH/relE 90/115-140*	GI		DK02	P11M2	2006b	90.6 kb	33 years	Hypermutator
Complete loss of GI	*RHH/relE 90/115-140*	GI		DK02	P40F2	2002	90.6 kb	29 years	
Complete loss of GI	*RHH/relE 90/115-140*	GI		DK02	P43M2	2002	90.6 kb	29 years	Hypermutator
Loss of one out of two GIs	*RHH/relE 90/115-140*	GI		IT11	P55M4	440	89.1 kb	0.79 year	
Partial loss of GI, TA remains	*RHH/relE 90/115-140*	GI		DK03	P62M4	All but one isolate	40 kb		
Partial loss of GI, TA remains	*RHH/relE 90/115-140*	GI		DK36	P77F4	All but 410, 410.1	50 bp	0.83 year	Integrase deleted
Duplication of GI	*xre/COG5654 & merR/PIN*	GI		DK26	P92F3	All			
Duplication of GI	*xre/COG5654 & merR/PIN*	GI		DK21	P22M4	181, 182		1.06 years	
Partial loss of GI, TA remains	*xre/COG5654 & merR/PIN*	GI		DK02	P14F1	1973	24 kb	>1 year	
Partial loss of GI, TA remains	*HEPN/MNT*	GI		DK02	P43M2	2002	5 kb	29 years	
Partial loss of GI, TA remains	*HEPN/MNT*	GI		DK02	P33F3	2003.a, 2003.b	>100 kb	30 years	Part of the CG also deleted
Partial loss of GI, TA remains	*HEPN/MNT*	GI		DK02	P80F1, P11M2	2002.b, 2006.b	18 kb	29 years	

*List of mutations observed in longitudinally sampled clone types and stop codons observed in clinical and environmental (env.) isolates. The location of a TA pair is given as core genome (CG) or on a genomic island (GI) and the mutation as the nucleotide change within the gene. For deletions, the estimated length in kb is noted, and for each mutation the length of infection of the clone type at sampling time. Stop codons were observed in three antitoxins that were sampled only once for that clone type from two patients and an environmental isolate. The toxins of the antitoxins with stop codons and an insertion were likely still functional, as their sequences were identical to those from other isolates. TA pairs that were identified by TAfinder but not verified by BLASTP analysis are highlighted with green*.

The TA loci were categorized by conserved domains, and for some as belonging to a (super-) family. Based on TAfinders classification there were six different toxin families/domains, and eight different antitoxin families/domains represented (and additionally three unverified toxins and four antitoxins). We named each TA pair according to the family or domain (Makarova et al., 2009) as identified by TAfinder, but note that these are not always identical to the conserved domains identified by BLASTP (Table S3), and protein length in amino acids. For each toxin and antitoxin family, 1–15 proteins were identified (Figure 1; Table 1). All but 12 protein sequences had a 100% identity match in GenBank (Table S3). The 12 unique sequences were given the accession numbers KY855415-KY855426. Alignments of the unique sequences to the reference sequences from TAfinder are available in the Supplementary Online Material (Data Sheet 1). Three TA systems previously identified in PAO1 were not annotated by TAfinder. When the protein sequences of these were analyzed in TAfinder, they shared 67% (HigAB, classified as Xre/RelE by TAfinder, category 1), 72% (ParDE, classified as PHD/RelE, category 1) and 53% (HicBA, category 1) identity with pairs recognized by TAfinder. This suggests that the chosen cut-off at 80% identity is somewhat conservative and leaves some TA systems unidentified, however, lowering this obviously increases the risk of including false positives.

Comparison of the genes flanking TA pairs showed that they were often highly conserved among clone types, and the GIs with a given TA pair were in most cases found in one or two locations in the genome (Tables S1, S3). Many of these insertion sites have already been identified as sites with “pathogenicity islands” (Liang et al., 2001; He et al., 2004; Qiu et al., 2006; Battle et al., 2008) and “regions of genomic plasticity” (Mathee et al., 2008; Table S3). BLASTP analyses of the GIs showed that they frequently harbored “mobility genes” such as transposases and integrases, suggesting that they are, or have been, mobile elements. The frequently observed PHD/RelE pair (category 1) is known to reside on the prophage Pf4 (Webb et al., 2004), which is important for biofilm development (Rice et al., 2009).

We commonly observed two TA pairs on the same GI, as marked in pink in Table S1. For example, the Xre/RelE 96/99 pair (category 2) was only found together with RHH/RelE 90/115-140 (category 1), while the latter was alone on a GI in 75% of the clone types. The AbrB/RelE system (category 1) was sometimes found in the place of the unverified system Xre/COG5654 208/210 (category 4), with the same flanking genes. This could suggest that the latter may also be a functional TA pair.

Not all TA pairs were found on contigs long enough to determine their insertion site in the genome and provide the full-length sequence of the GI. This was often the case when two GIs of the same type were present, suggesting that the presence of highly similar regions hinders *de novo* genome assembly. The insertion sites of an “orphan” GI (TA systems found on a contig with no link to the core genome) could often be inferred when specific insertion sites for that GI had been identified for other clone types: If the known insertion sites were found on separate contigs flanked by GI-specific genes this was assumed to be the insertion site. In a few cases no insertion site was identified. It cannot be ruled out that these TA pairs were found on plasmids, however the sequencing coverage of these regions were comparable to the average of the isolates, suggesting that if so they would be low-copy plasmids.

### TA loci acquire few mutations during infection

In the longitudinal samples, we only observed two mutational events in core genome TA loci during infection; the complete loss of a pair as part of a large >40 kb deletion in DK13, and a non-synonymous SNP in a toxin in one isolate of DK1 (Table 2). All samples of DK21 from one patient had *ca*. 40 kb insertion disrupting the Xre/COG5654 pair (category 4). Further, three clone types, that were sampled only once from a patient and from the environment, harbored stop codons or an insertion in toxin or antitoxin genes in the core genome (DK23, DK49, and MG7), and one had an apparent deletion and insertion covering the HigAB pair (DK28, category 1; Table 2).

### GIs with TA systems may be mobile but show limited transmission

The mobility of some GIs was supported by the finding of a circular GI in one patient: the GI comprised an individual contig, where the end-genes were found in the middle, and the core genome flanking genes were found next to each other on a separate contig (RHH/RelE 90/115-140, DK11, category 1). Further, two GIs with unverified TA systems were found to double their copy number during infection (Table 2). However, we found no evidence for horizontal transmission of GIs with TA systems between clone types within patients. When clone types co-occurring in a patient shared a GI type and TA system, the sequences had >50 SNPs, which would not be expected if they had been transmitted recently, given the average rate of mutation of 2.5 SNPs/year (Marvig et al., 2013).

Transmission between patients is evident for three clone types, DK6, DK15 and DK26 (Marvig et al., 2015b; Table S1), where the GIs with TA systems were identical for DK6 and DK15. For DK26, isolates from one patient (P99F4) showed an apparent acquisition of a >212 kb GI not found in other isolates, with a TA pair (COG5606/RelE 111/125, category 3) found in two other clone types. In another patient with DK26 (P92F3), there was a duplication of an already present GI with an unverified TA pair, as mentioned above (Table 2; Table S1).

### GIs with TA systems are more frequently lost than gained during infection

Complete or partial loss of GIs carrying TA systems during infection was observed 15 times in the sample population, in 7 out of 52 clone types. One isolate of IT11 lost a GI, that is present in two copies in the other isolates from the patient. In four isolates of the transmissible DK2 clone type, we observed the complete excision of the most common GI (with RHH/RelE 90/115-140, category 1) in four independent events, after 29 (three isolates) and 33 (one isolate) years of infection, respectively. This GI was also lost in one isolate of DK44. Partial loss of a GI including two unverified TA systems occurred once in DK26. However, there were eight events in four clonetypes, where TA systems remained despite partial loss of a GI, in an isolate of DK3, an isolate of DK24, two isolates of DK36 and four isolates of DK2 (unverified category 4 pairs; Table 2).

To estimate the rate of loss and gain of GIs with TA systems on an ecological time scale, we used the nine clone types that had been independently acquired by multiple patients from the environment. For these we had information on the number of SNPs in the core genome differentiating them (Marvig et al., 2015b). As an example, for clone type DK6, the isolates most closely related to each other, sampled from two patients, are separated by 421 SNPs (~168 years). Three GIs are only present in one of the DK6 lines giving an estimated rate of 0.033 losses or gains of GIs per year, or one event per 30.4 years. This gives us 11 independent estimates of gain and loss of GIs, as two clone types contributed two data points each (Table S2). We recorded between 0 and 6 events that had occurred over an estimated 21.6–201.6 years. This lead to on average one loss or gain every 52.69 ± 42.21 years std. dev. and a range between an event every 9.9–151.6 years (excluding the data point with 0 events).

### Correlation between TA systems on GIs and the ability to establish chronic infection

There was no significant difference in the number of TA systems of a clone type and its ability to establish a chronic infection, as defined in the Materials and Methods (One-Way ANOVA, *df* = 3; 33.67; *F* = 0.65, *p* = 0.59). The most commonly occurring RHH/RelE 90/115-140 TA system (category 1) residing on GIs was, however, found more often in clinical isolates compared to environmental isolates (70% of first clinical isolates compared to 33% of environmental, χ^2^ = 4.4, *df* = 1, *p* < 0.05, with the caveat of a small sample size of environmental isolates). Prevalence of the RHH/RelE 90/115-140 TA system across different categories of clinical isolates was similar, ranging from 64 to 75%.

## Discussion

We present, to our knowledge, the largest study of intraspecies diversity of type II TA systems, in clinical isolates of *P. aeruginosa*. The systems were identified computationally with the program TAfinder and subsequently verified by determination of conserved domains. The pairs were split into four categories of TA systems based on the likelihood that these are functional TA systems. Such variation highlights the inherent risk of relying on a computational approach, and emphasizes the need for experimental validation. However, the aim of our study is a broad analysis of a large collection that will serve as a precursor to experimental work, and therefore we include all genes that are already verified or identified as novel putative TA systems. In support of this stance we found that for the two experimentally confirmed systems, the HigB toxin and the HicB antitoxin did not have specific hits as such in the BLASTP analysis. We also note that more toxins were verified than antitoxins, perhaps suggesting a greater diversity in the latter.

We clearly distinguish between TA systems found in the core genome and on GIs. The core genome harbors four TA pairs, while 22 other pairs are on GIs and variably found across isolates, responsible for the diversity of TA systems in the population. It is unclear if the core genome TA systems have been “domesticated” from mobile elements or have evolved within *P. aeruginosa*, but the former seems possible as they were also found in other non-Pseudomonas species (Table S3). We do not find a correlation between the number of TA systems an isolate harbors and its ability to establish as a chronic infection.

The function of TA systems in *P. aeruginosa*, and in particular in infection, is relatively unexplored. Recently, two pairs were experimentally confirmed to be TA systems, the core genome HigAB pair (Wood and Wood, 2016) and the HicBA pair found on a GI (Li et al., 2016b). For HigAB, deletion of the antitoxin gene *higA* results in reduced growth, swarming and biofilm formation, and a decrease in production of pyocyanin and pyochelin, all of which are phenotypes affecting virulence. The HicBA pair was found to have a bacteriostatic effect and cause cell aggregation. However, a knock-out of *hicBA* did not affect biofilm formation or infectivity in an acute mouse-infection model (Li et al., 2016b). Contrary to our study, the prevalence reported in the Li et al. (2016b) study of the HicBA (33% of 996 isolates screened) was much higher than that observed here (4%, of which only two toxins out of four appear to have an antitoxin), for unknown reasons. On the other hand, the HigAB (category 1) and the RHH/RelE (category 2) core genome pairs and one GI TA pair (PHD/RelE or ParDE, category 1) have been found to be expressed in CF sputum, suggesting they may play a role in infection (Williams et al., 2011).

### GI-residing and core genome TA systems are stable in clinical and environmental isolates

We observed few mutations in the core genome TA loci during infection. Notably, the few isolates with stop-codons and an insertion in core genome antitoxins were not successful at establishing infection, as they were either found as clone types only sampled once or from an environmental isolate. Two of these, interestingly, had the same mutation introducing a stop codon (Table S3). This corresponds with other studies (Mine et al., 2009) where TA loci are not acquiring loss of function mutations and, therefore, are not selected against but maintained as selfish elements or for other physiological cellular processes.

Complete or partial loss of GIs during infection was rare, suggesting that they are beneficial pathogenicity islands facilitating host colonization (He et al., 2004) and/or that TA systems are successful at stabilizing them. In six out of 15 cases an entire GI was lost, and because these were found in the same locations as documented ICEs this may represent active excision of mobile elements. Excision may be a way for the ICE to “jump ship” in search of a new host in the event of cell damage. This was seen in *Pseudomonas knackmussi* where the fraction of the cell population in an ICE “transfer-competent” state increased in the presence of reactive oxygen species and cell damage (Reinhard and van der Meer, 2014). In eight of the nine partial losses of GIs, the TA systems were intact, which could indicate that TAs can stabilize a part if not all of a GI. Even though pathogenicity islands are well-documented to be beneficial during infection (He et al., 2004; Battle et al., 2008), horizontal transfer of these or other GIs was not observed between clone types within patients. This may reflect that infecting isolates already have the GIs they need or that spatial segregation of co-occurring clone types within the lung and sinus environment limits the possibility for transfer.

To evaluate the stability of GIs in patients, we used the variation in TA-bearing GIs within clone types that had been independently acquired from the environment to estimate the rate of loss and gain of these on an ecological timescale. This gave an estimate of one loss or gain of a GI every 53 years. There are some obvious caveats with this estimate: the number of SNPs in the core genome only provides a rough estimate of the time since the last common ancestor, and GIs may have been lost and acquired repeatedly in the time between sampling of the compared isolates. That said, the estimate of one event per 53 years is in line with what we observe in the clinical isolates where loss occurs rarely: in the longest sampled clone type, DK2, we recorded losses only four times after 29 and 33 years of infection (Table 2; three of these deletions occurred in isolates with an increased mutation rate, so-called hypermutators with a mutation rate >3.6 times higher than the other isolates (Marvig et al., 2013). Comparable stability of GIs in the environment and in patients suggests that they are not only maintained for pathogenesis, and further supports a stabilizing effect of TAs.

### TA systems and bacterial pathogenesis

Our findings show that within *P. aeruginosa* there is no correlation between an incremental number of TA systems and the ability to establish a chronic infection. Previous work in *S. typhimurium* and *E. coli* have shown an association between certain TA systems as well as a correlation between the cumulative number of TA systems and level of persistence (Maisonneuve et al., 2011; Helaine and Kugelberg, 2014; Helaine et al., 2014) and there is a strong correlation between the two across eight *Pseudomonas* species (Vogwill et al., 2016). They have also been implicated in inducing biofilm formation in *E. coli* and enhancing host virulence in *H. influenza, Mycobacterium tuberculosis*, and *S. typhimuriun* (Lobato-Marquez et al., 2015), and inducing persistence in the development of recalcitrant infections in *P. aeruginosa* (Mulcahy et al., 2010).

We find that GIs with the RHH/RelE 90/115-140 TA pair were found significantly more frequently in clinical than environmental isolates, supporting a correlation between infectivity and these TA-bearing GIs. They were found at two insertion hotspots where multiple pathogenicity islands have been described (in the intergenic regions PA0976//PA0988 and PA4541//PA4542; Table S3), from which some genes have been shown to affect virulence in infection models (He et al., 2004; Battle et al., 2008). The GIs sharing these insertion sites are likely mosaic in nature, with recombination occurring within and between isolates, and even between species (Larbig et al., 2002; He et al., 2004). A similar pattern of some specific TAs being found more often in pathogenic isolates has been observed in the related *Pseudomonas putida* (Molina et al., 2016).

Our results suggest that some TA systems in *P. aeruginosa* may serve in the maintenance of GIs and do not appear to play a role in persistence or may not even be functional. *P. aeruginosa* is highly versatile and ubiquitous in the environment, as well as an opportunistic pathogen (Stover et al., 2000). Therefore, it may rely on a baseline number of TA systems, to mediate stress responses while colonizing/infecting a diverse range of environments and host types with variable abiotic conditions. If so, harboring additional TA systems may not provide greater benefits to strains, specifically in infection compared to other environments, and the core genome TA systems could be all that is needed.

## Conclusions

Screening for the prevalence of TA systems has become a popular pursuit (Pandey and Gerdes, 2005; Sevin and Barloy-Hubler, 2007; Leplae et al., 2011; Shao et al., 2011), as chromosomal type II TA systems were discovered to play a role in the regulation of cell metabolism, particularly for persister cell formation (Kim and Wood, 2010; Maisonneuve et al., 2011; Fernandez-Garcia et al., 2016). Although extensive research has explored the numerous biological functions proposed for chromosomal TA systems, their role as selfish, stabilizing elements, and in bacterial stress response is still open to debate. While our work highlights the importance of localizing, rather than simply counting TA systems it should be noted that their location in the genome does not limit them to specified functions. In addition, their potential function as stress response elements does not exclude stabilization properties, emphasizing that studies must be cautious in assigning single functions. While the majority of TA systems identified here remain to be confirmed experimentally, our study is the first to track putative TA systems over time in a clinical system and we show they are remarkably stable, both when found in the core genome and on GIs. A key finding of this study is the discovery of a set of core TAs, irrespective of where the isolates were sampled. These may play a role in the regulatory network of *P. aeruginosa*; enhancing survival across fluctuating environmental conditions and during adaptation in a host. The next step will be to experimentally test such a regulatory role, particularly in pathogenic *P. aeruginosa* isolates in the development of persister cells contributing to recalcitrant infections.

## Ethics statement

All isolates came from the Department of Clinical Microbiology, Rigshospitalet, Copenhagen, where they had been isolated from CF patients treated at the Copenhagen CF clinic. The use of samples was approved by the local ethics committee at the Capital Region of Denmark Region Hovedstaden: registration number H-4-2015-FSP. All patients have given informed consent. In patients under 18 years of age, informed consent was obtained from their parents. All patient samples have been anonymized prior to any experimental use or analysis.

## Author contributions

MG and SA designed the study; MG, SA, and BP analyzed the data; HJ and SM acquired the samples and patient and genomic data; All authors contributed to the interpretation of the data, the writing of the manuscript, approved the final version and agree to be held accountable for the accuracy and integrity of the work.

### Conflict of interest statement

The authors declare that the research was conducted in the absence of any commercial or financial relationships that could be construed as a potential conflict of interest.

## Supplementary material

Table S1 Overview of TA prevalence in *P. aeruginosa* isolates. For each isolate is stated whether it comes from a CF patient or the environment, its clone type, the total number of TA systems (including four core genome pairs), the isolate number (as listed in Marvig et al., 2015b), the clone type's persistence in the patient, whether the clone type had been transmitted between patients and the number of contigs in the *de novo* assembled genome. Subsequently all TA pairs found on genomic islands are listed, and their prevalence in an isolate marked followed by the observed or predicted location of the genomic island. TA pairs marked in pink within an isolate are found on the same GI. Columns in green show TA pairs identified by TAfinder but not verified by BLASTP analysis (category 4 pairs).

Table S2 Estimation of the frequency of loss and gain of GIs with TA pairs in the environment. Some clonetypes found in multiple CF patients had been independently acquired from the environment. We identified the lowest possible number of events of loss or gain of GIs between two isolates of the same clone type as the difference in GIs, and estimated the time since the most recent common ancestor by the number of SNPs in the whole genome (Marvig et al., 2015b) and an average rate of 2.5 SNPs accumulating per year in the CF environment (Yang et al., 2011). From this we calculated the frequency in years of gain and loss of GIs. Below are comparisons with the three environmental isolates that shared clonetypes with the clinical isolates. These were not included in the analyses as the sequence coverage was lower and hence the calculated SNPs may be unreliable.

Table S3 Extended overview of TA diversity. This table adds to Table 1 by including the location in the genome of TA pairs and that of the genomic islands on which they reside, and where these have previously been described. TA names in green show pairs identified by TAfinder but not verified by BLASTP analyses. Conserved domains of TAs found by unrestricted BLASTP analyses are listed, yellow mark a specific hit and white an unspecific hit (see SOM). When these could not be verified, BLASTP analyses restricted to toxin or antitoxin was performed and the % shared identity to putative TAs is listed (blue highlight, see SOM). Domains without a match by unrestricted BLASTP analyses but predicted to be likely TAs by Makarova et al. (2009) are highlighted in purple. The pairs are classified as category 1-4 dependent on the presence of conserved T and A domains. The number of unique protein sequences identified in the study is shown, and if these were identical to previously identified sequences the GenBank accession number is listed. Twelve sequences did not have a 100% identity match and were submitted to GenBank (accession numbers KY855415-KY855426). For each pair it is listed which species they are also found in, as pairs. For all but two core genome pairs, Xre/GNAT and Xre/RelE (HigAB), three alternative TA “host species” are listed in the non-exhaustive list, as well as their % shared identity with the pair of our study.

Data Sheet 1 Supporting online material (Data Sheet 1): Alignments for each toxin and antitoxin, with the unique sequence(s) and the reference from TAfinder, denoted as “ref” and by the acquisition number from GenBank. Alignments are followed by the results for conserved domains from an unrestricted BLASTP search, or the top hits from a BLASTP search with Entrez Query restricted to “toxin” or “antitoxin.” In two instances neither gave a significant hit (COG5654 251 & 210).
