# Supplementary material for: Diversity, Prevalence, and Longitudinal Occurrence of Type II Toxin-Antitoxin Systems of Pseudomonas aeruginosa Infecting Cystic Fibrosis Lungs
Source: Front Microbiol. 2017 Jun 23;8:1180. doi: 10.3389/fmicb.2017.01180 (PMC5481352; doi:10.3389/fmicb.2017.01180)
Supplement: Data Sheet 1 — Supporting online material (Data Sheet 1): Alignments for each toxin and antitoxin, with the unique sequence(s) and the reference from TAfinder, denoted as “ref” and by the acquisition number from GenBank. Alignments are followed by the results for conserved domains from an unrestricted BLASTP search, or the top hits from a BLASTP search with Entrez Query restricted to “toxin” or “antitoxin.” In two instances neither gave a significant hit (COG5654 251 & 210). [file DataSheet1.DOCX]

**Supporting online material**

Alignments for each toxin and antitoxin, with the unique sequence(s) and the reference from TAfinder, denoted as “Ref” and by the acquisition number from GenBank. Alignments are followed by the results for conserved domains from an unrestricted BLASTP search, or the top hits from a BLASTP search with Entrez Query restricted to “toxin” or “antitoxin”. In two instances neither gave a significant hit (COG5654 251 & 210).

RHH/relE 75/93

Antitoxin discovered by Pandey & Gerdes 2005.


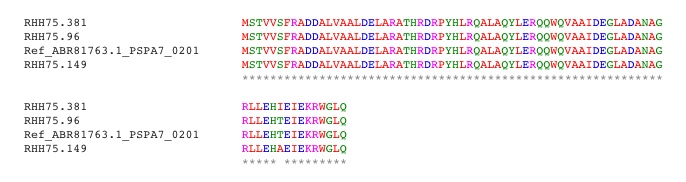


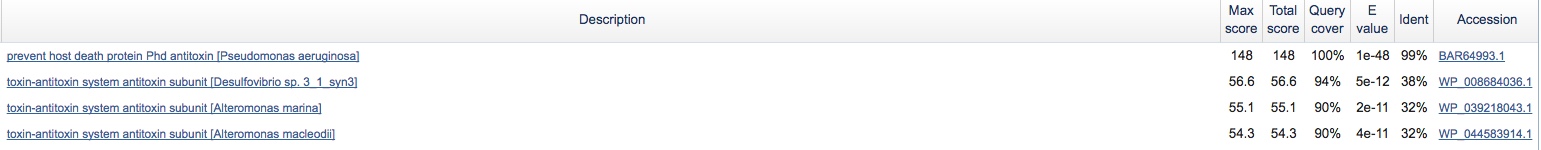


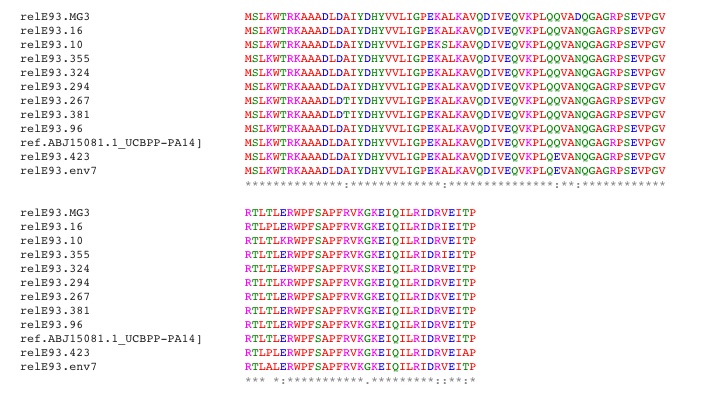


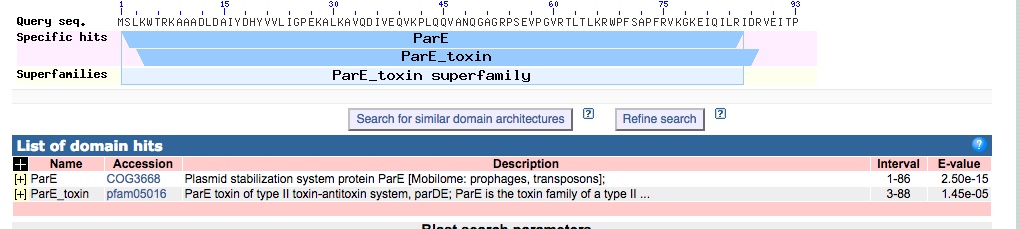


xre/COG5654 122/251


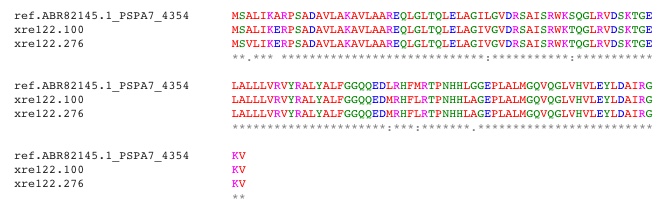


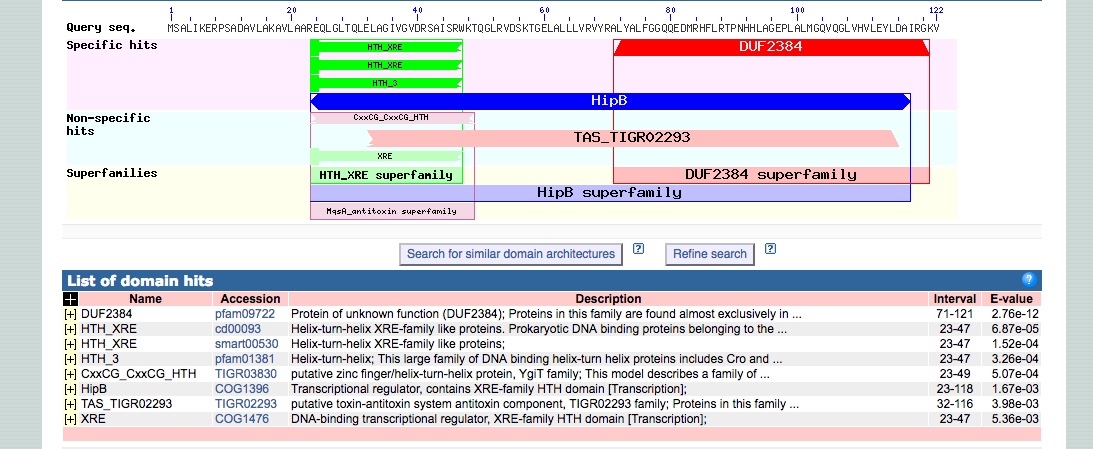


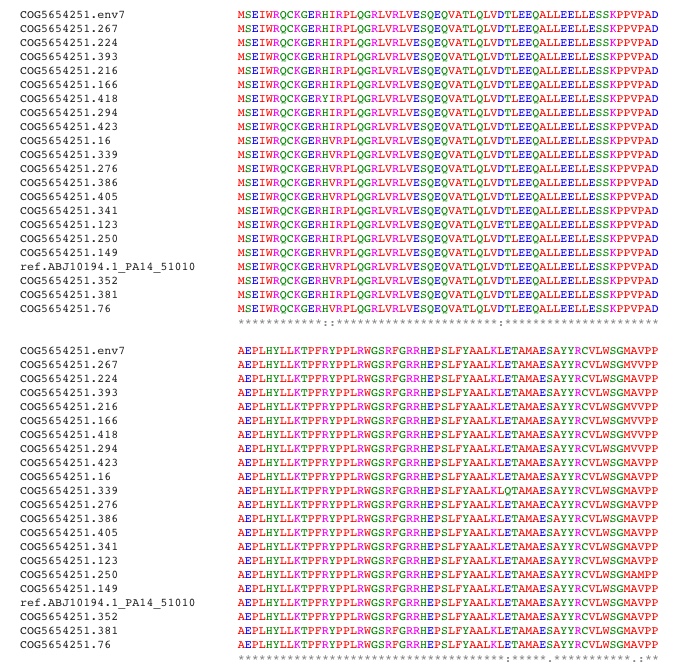


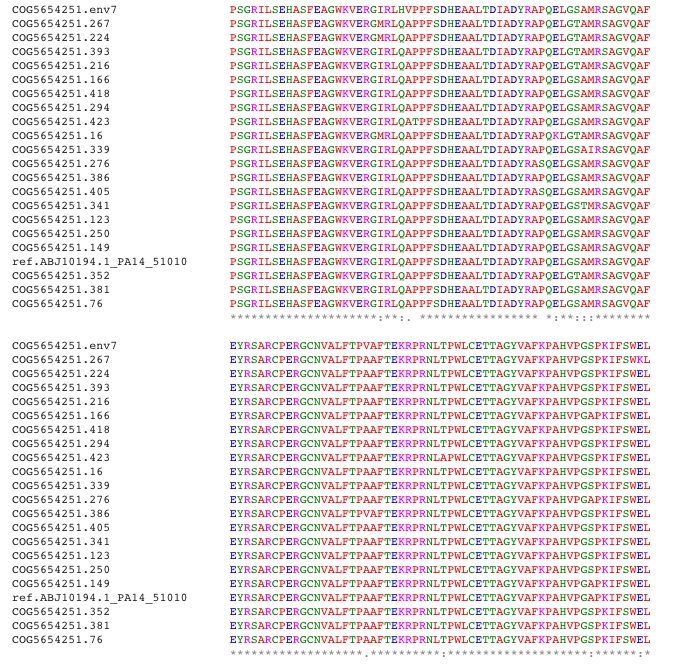


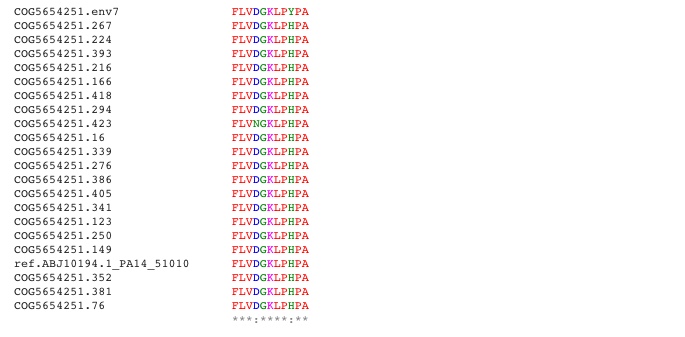


xre/GNAT 282-284/189 - unverified


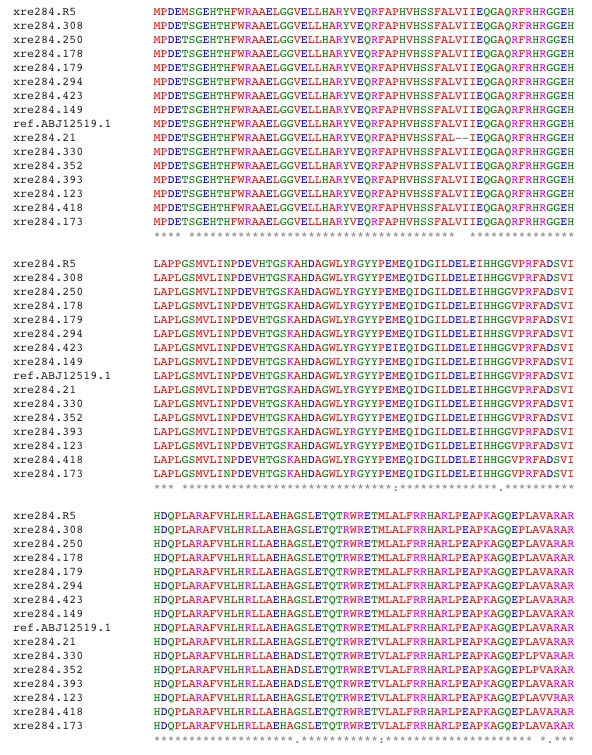

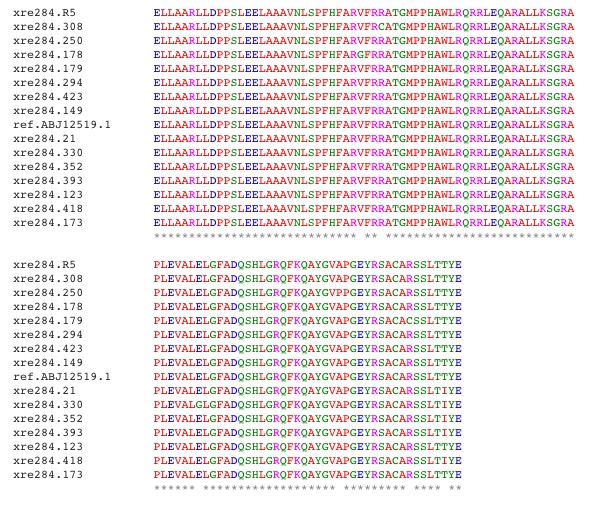


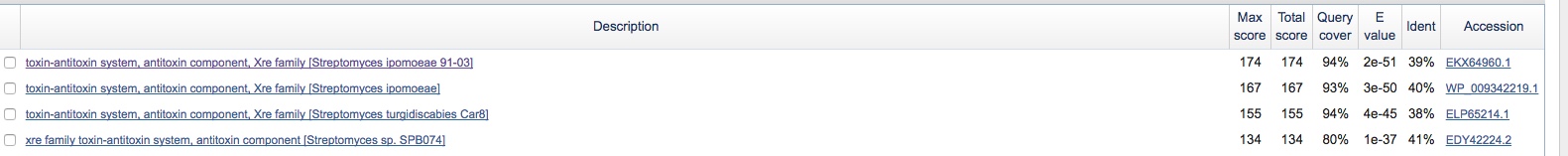


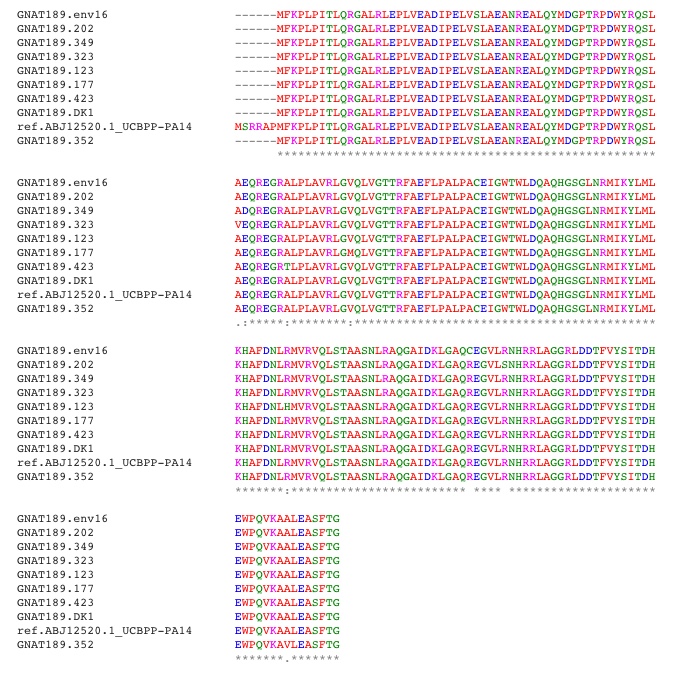


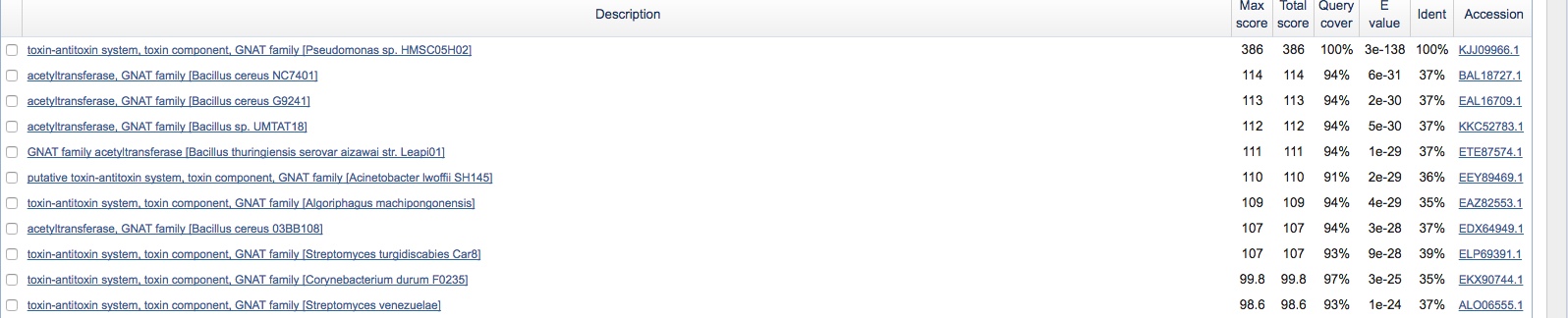


xre/PIN 184/192


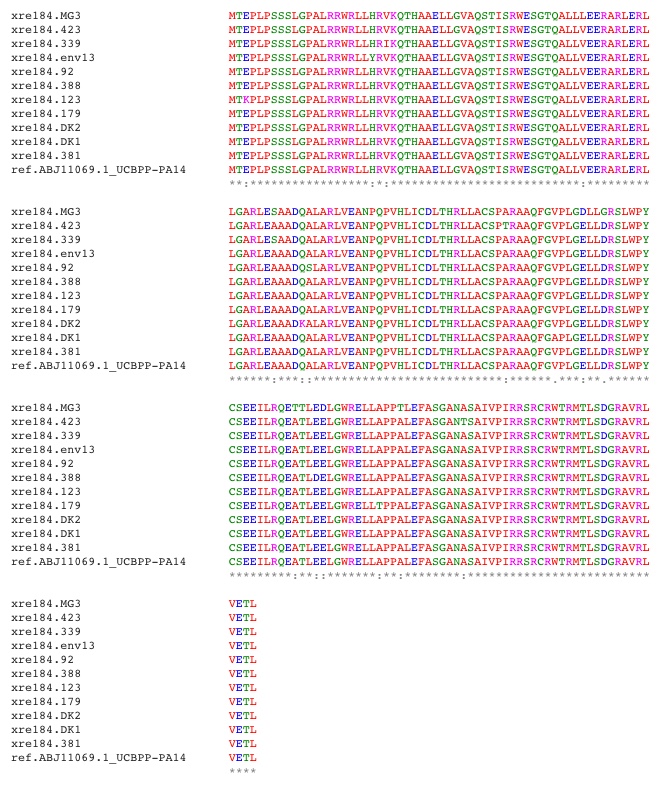


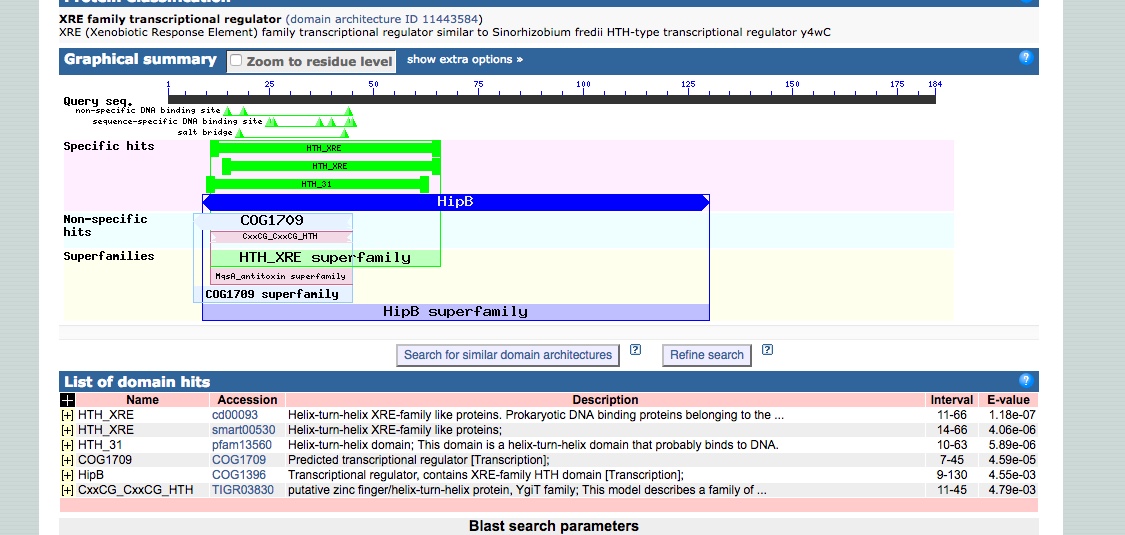


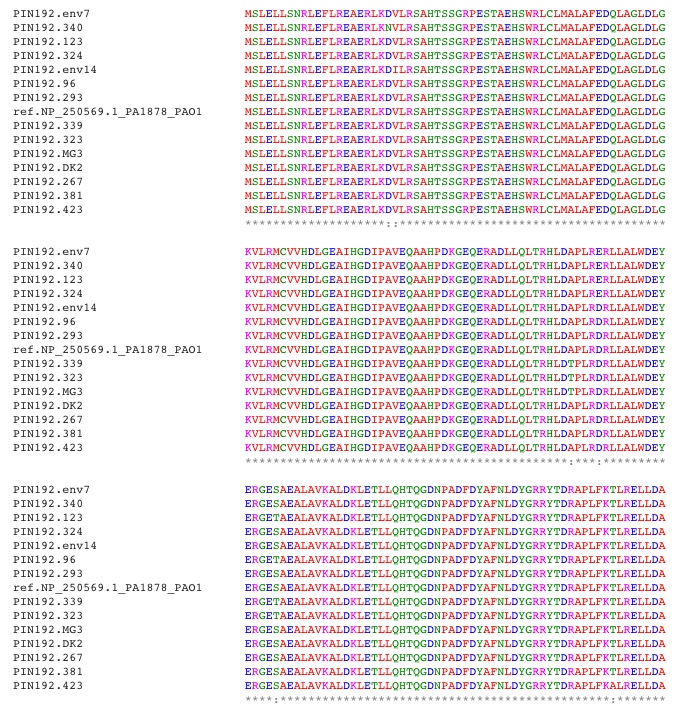


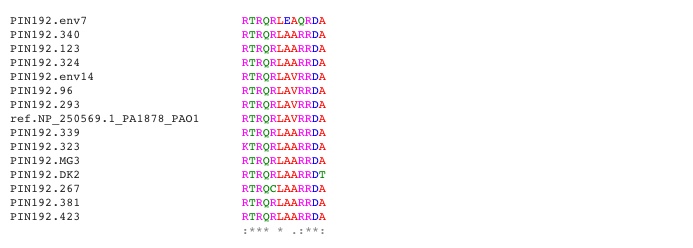


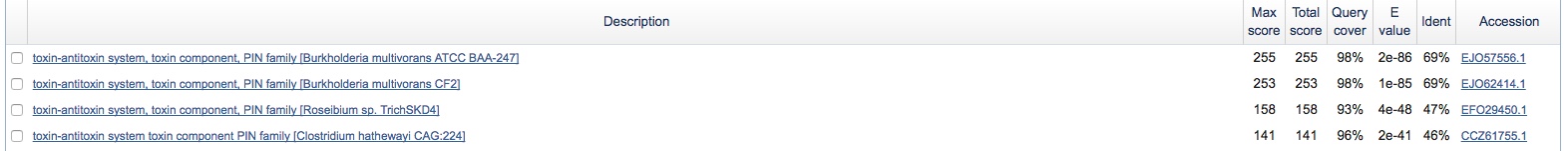


xre/relE (higAB) 101/89-92


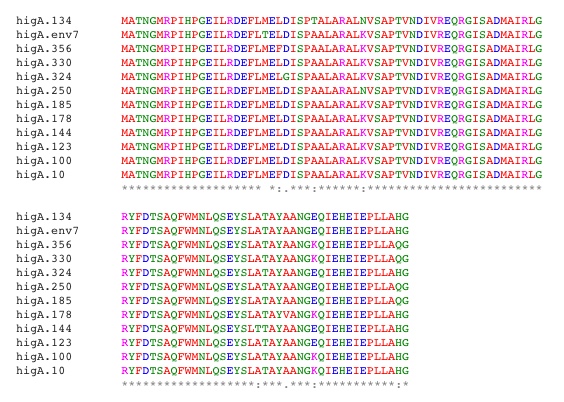


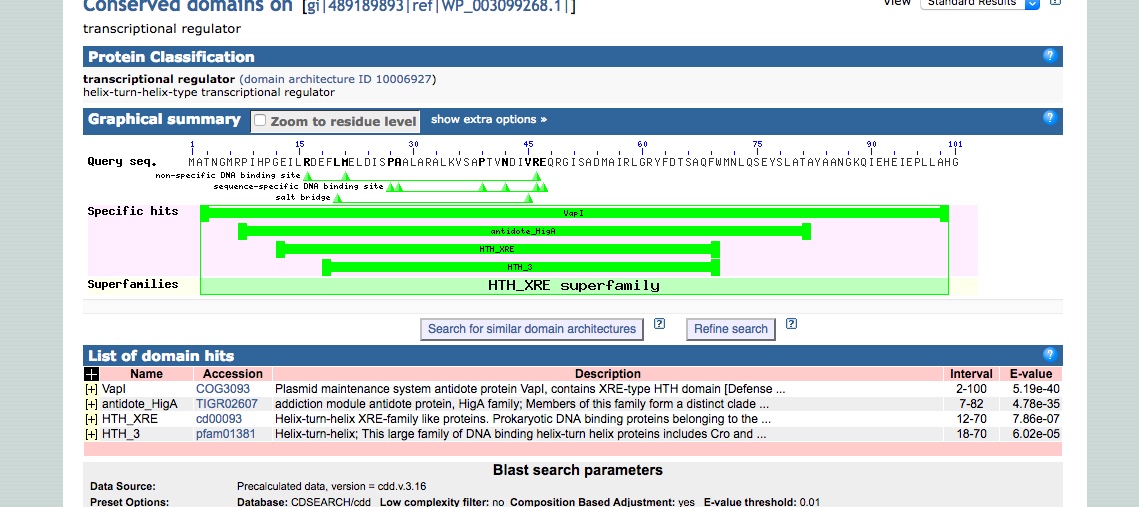


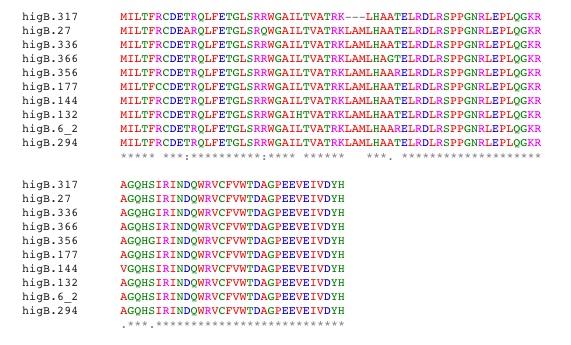


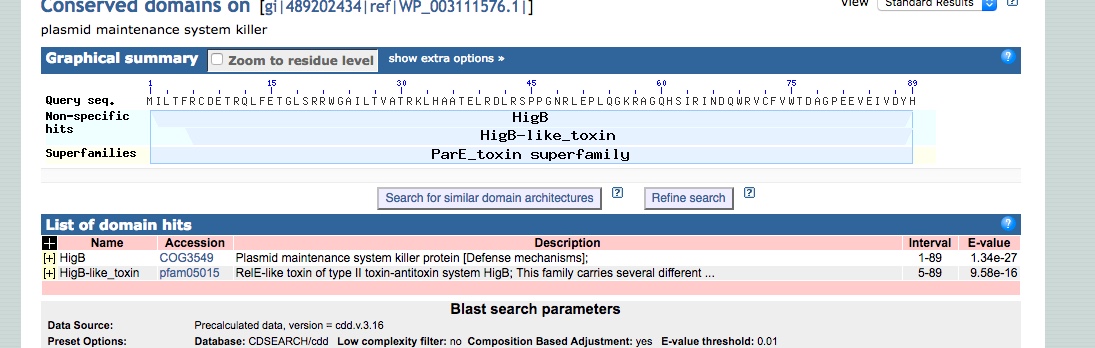


RHH/relE 90/115-140


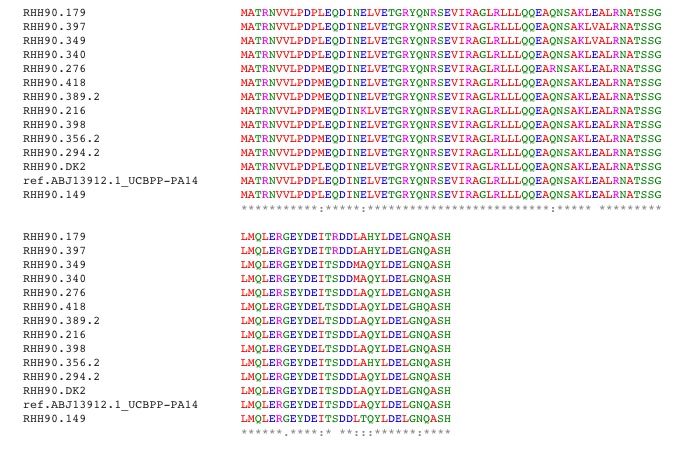


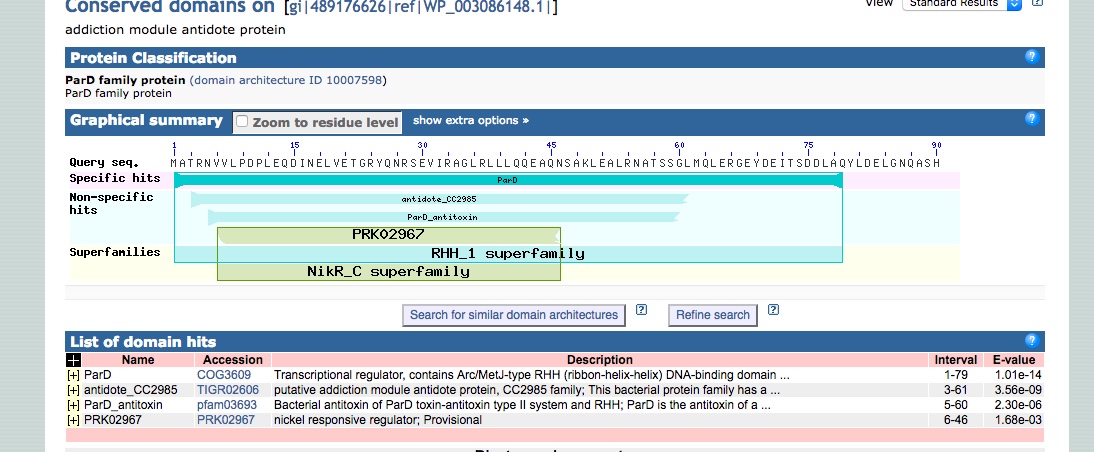


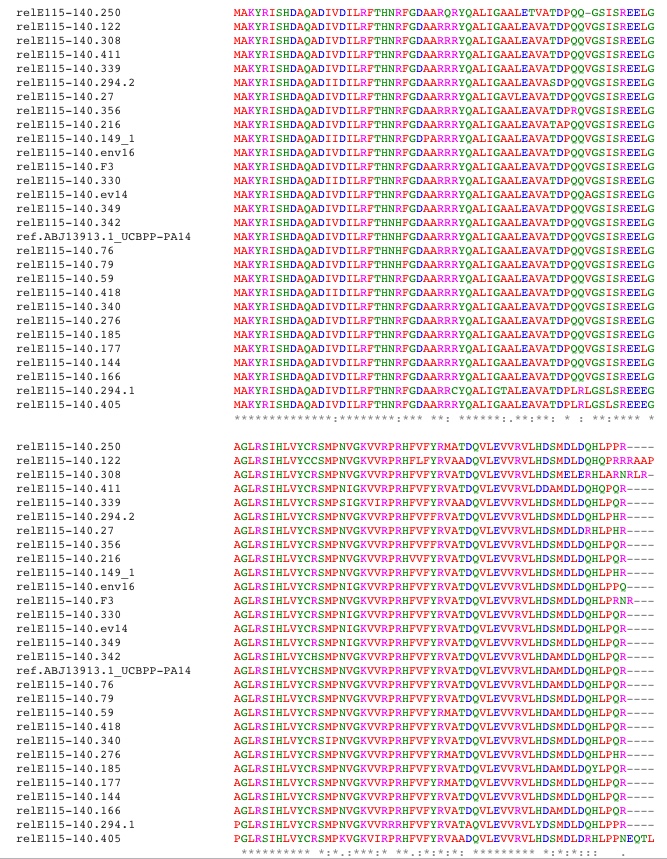


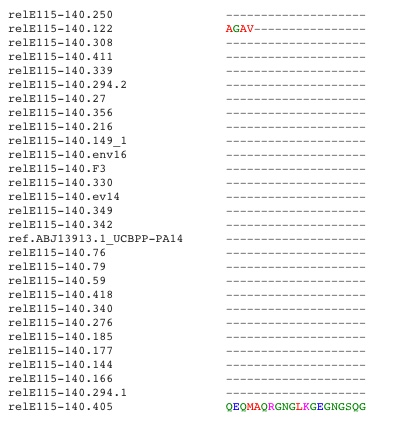


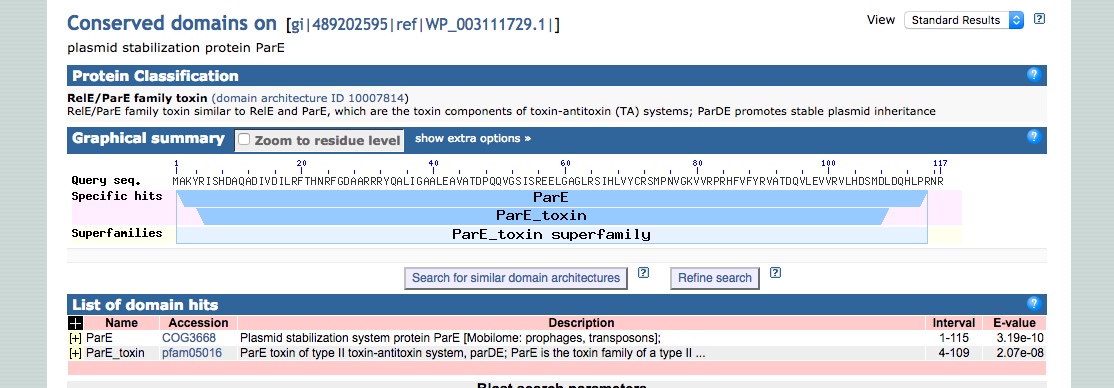


xre/COG5654 208/210 - unverified
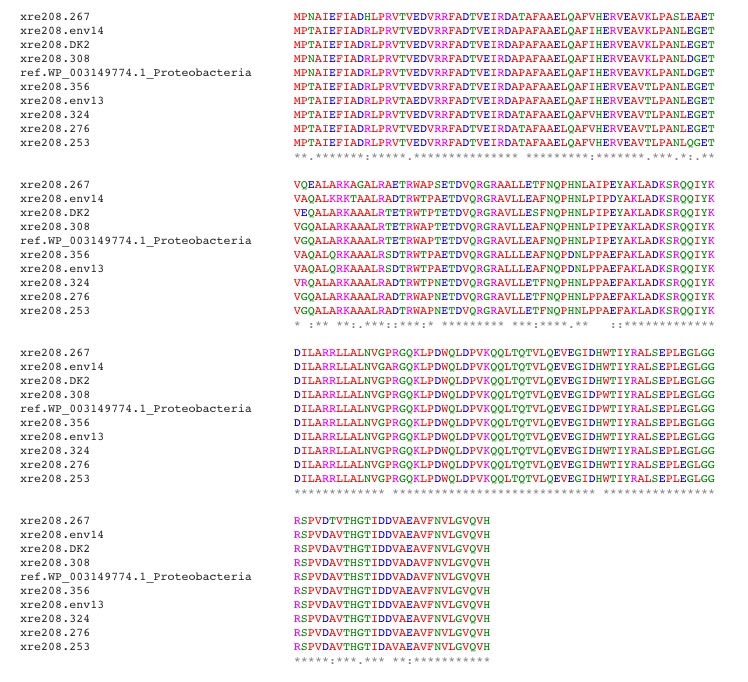


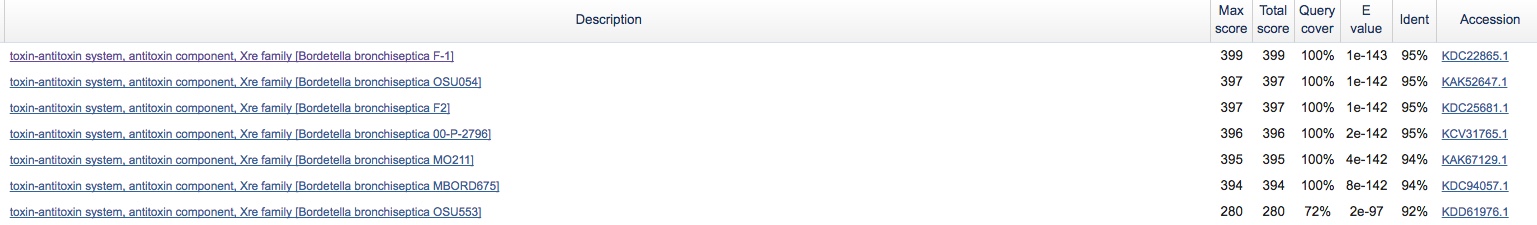


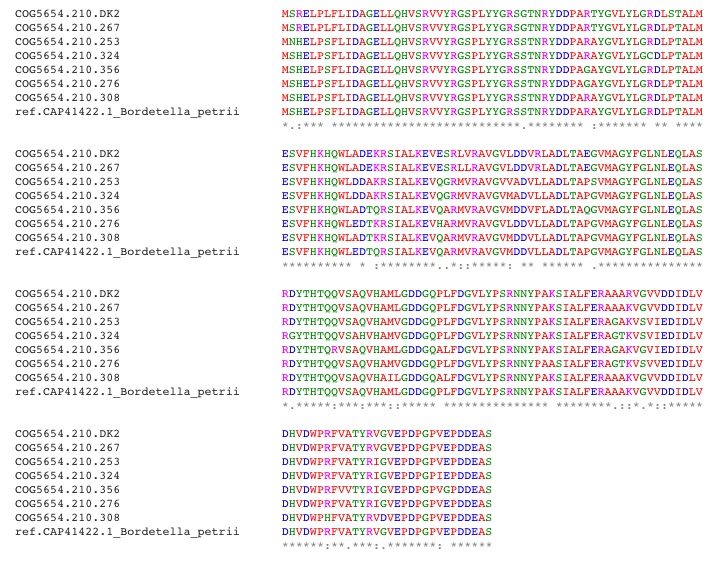


merR/PIN 156/191 - unverified


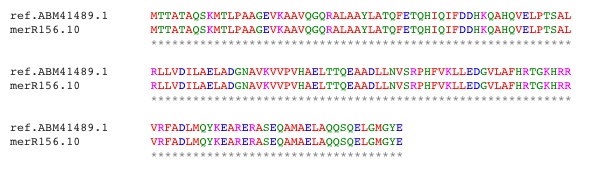


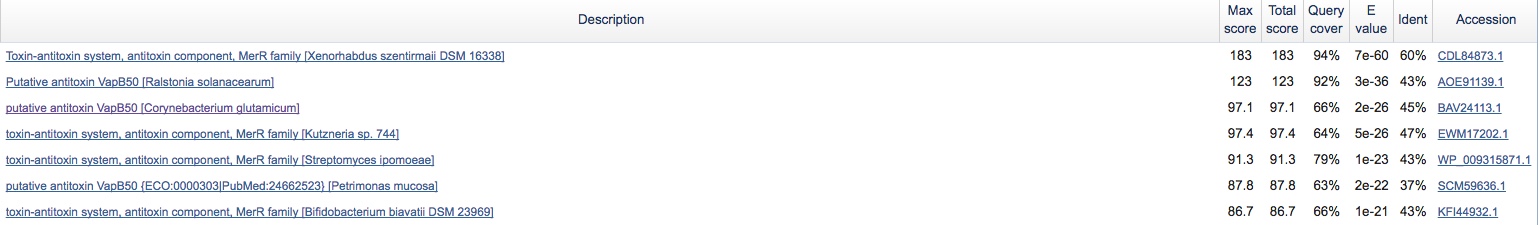


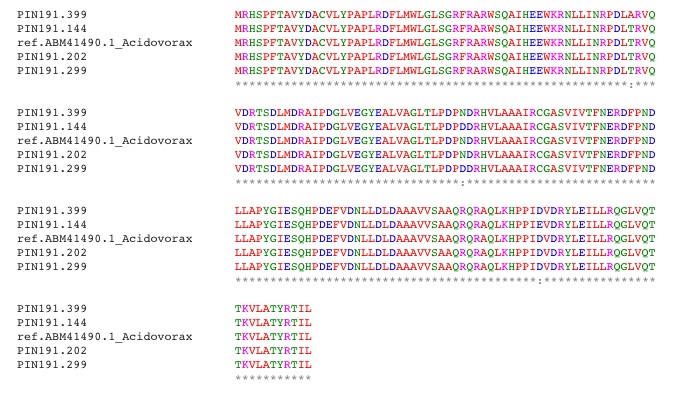


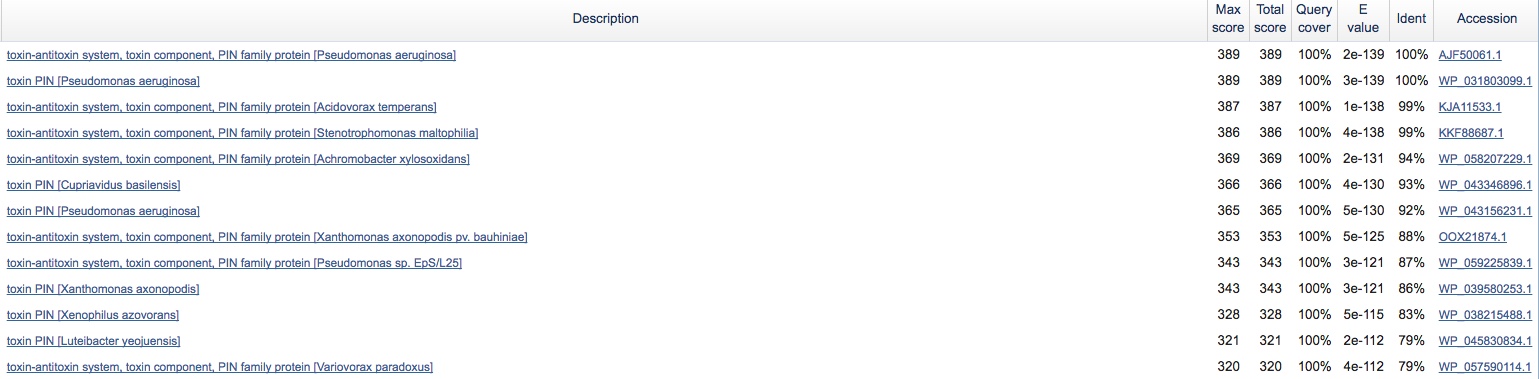


PHD/relE (parDE) 83/115


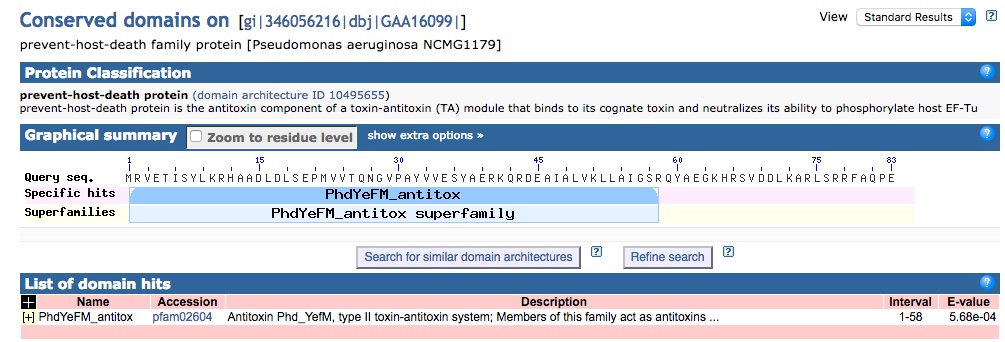


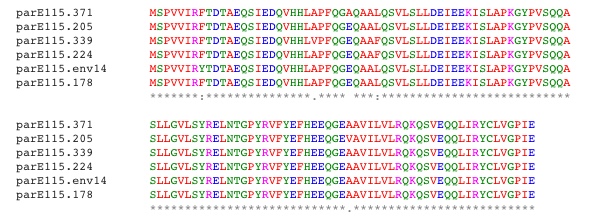


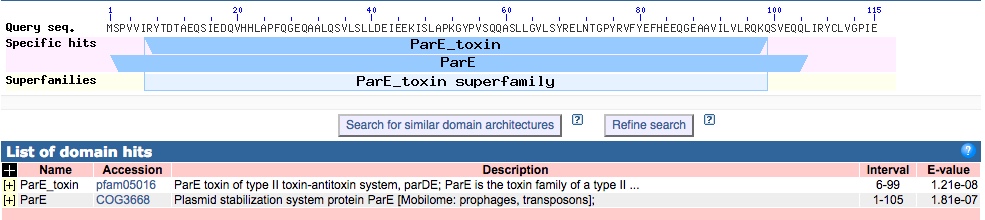


xre/relE 96/99


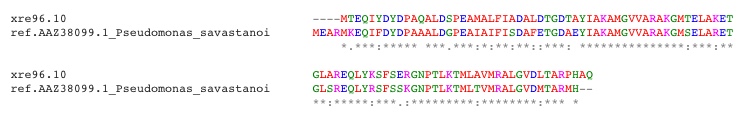


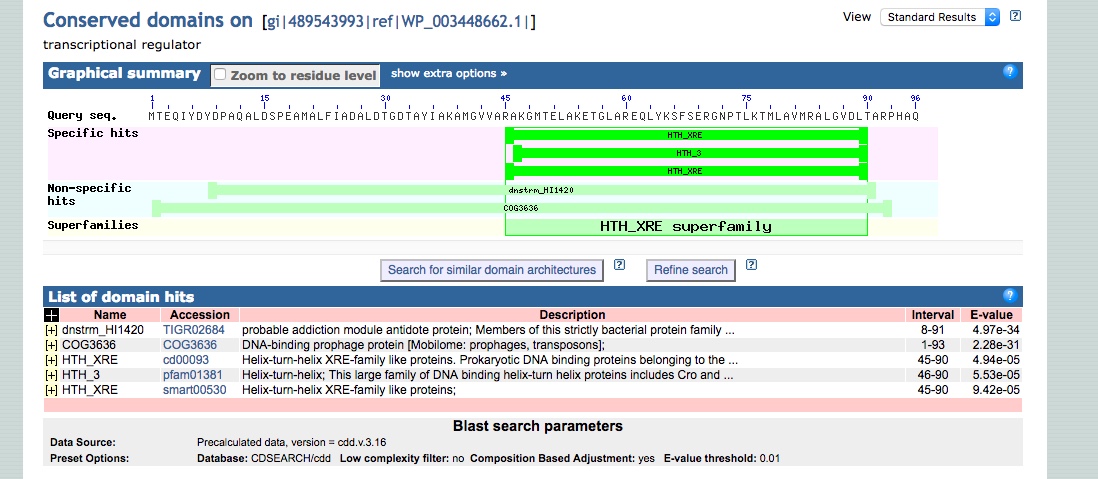


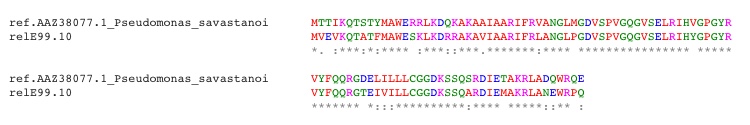


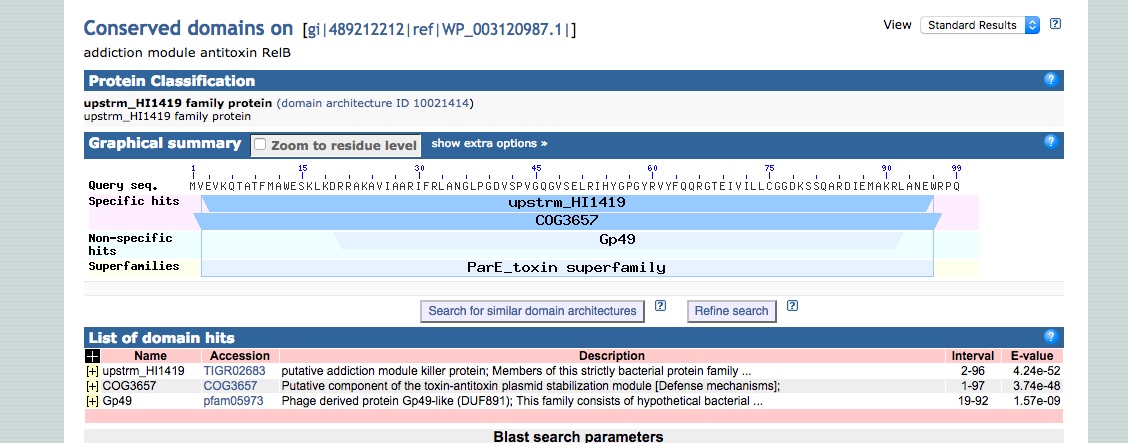


abrB/relE 105-123/152-154


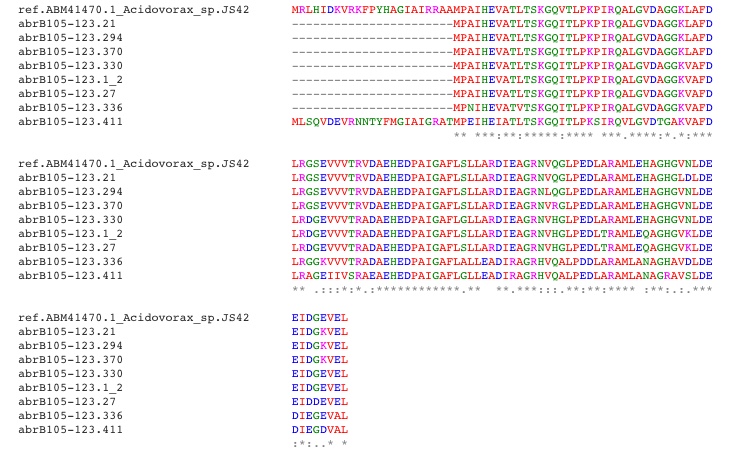


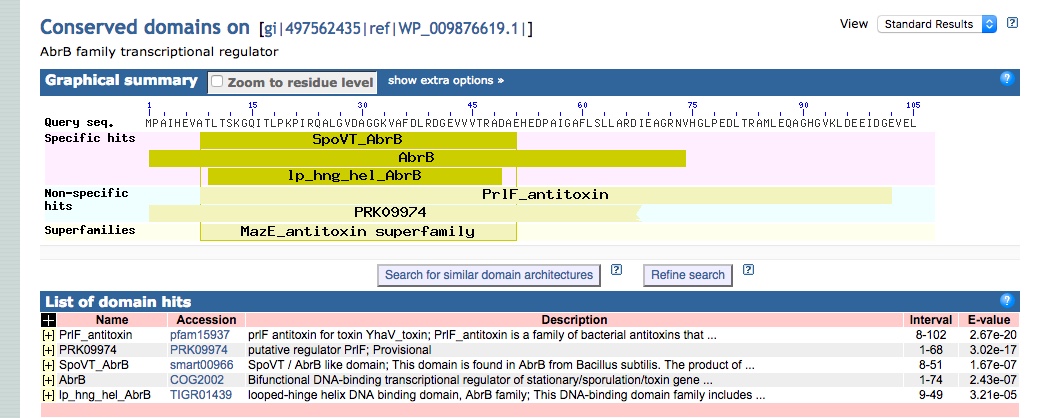


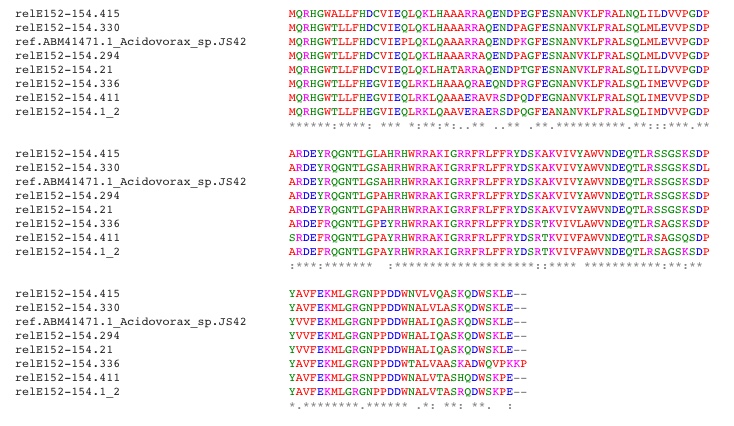


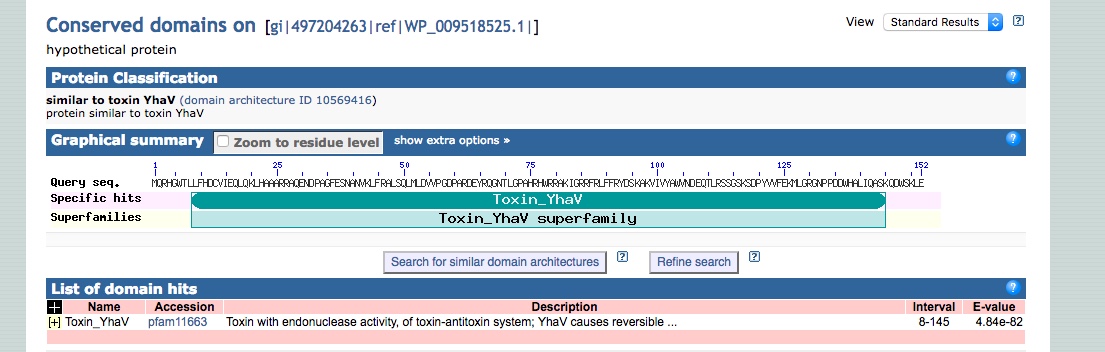


xre/relE 100/126-127


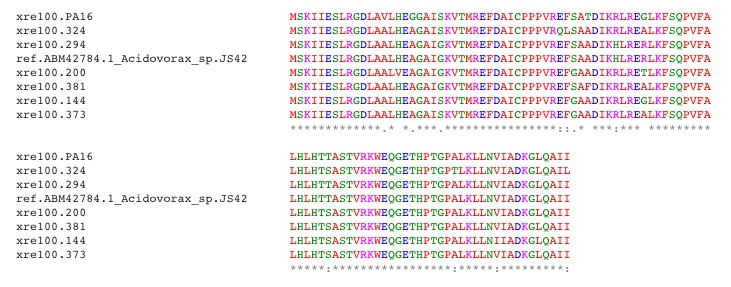


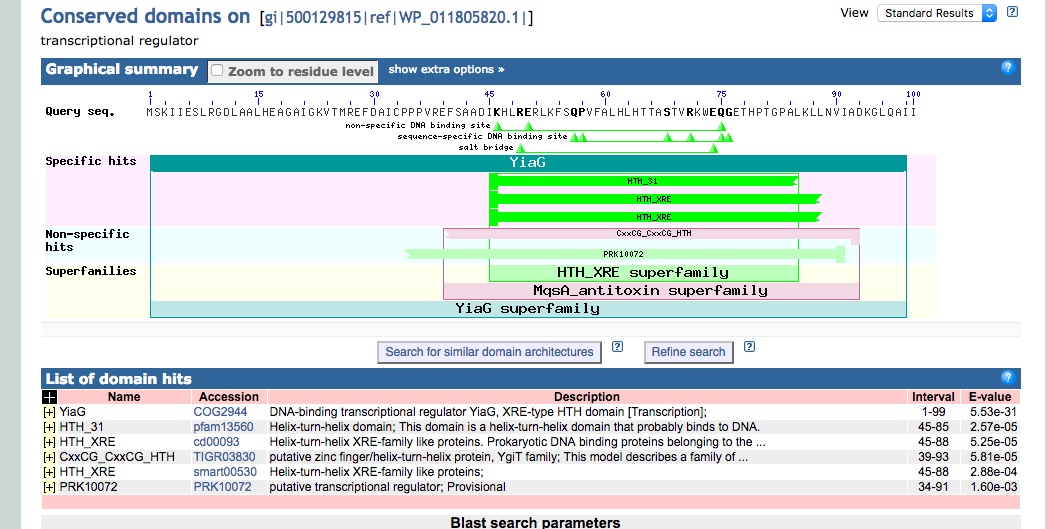


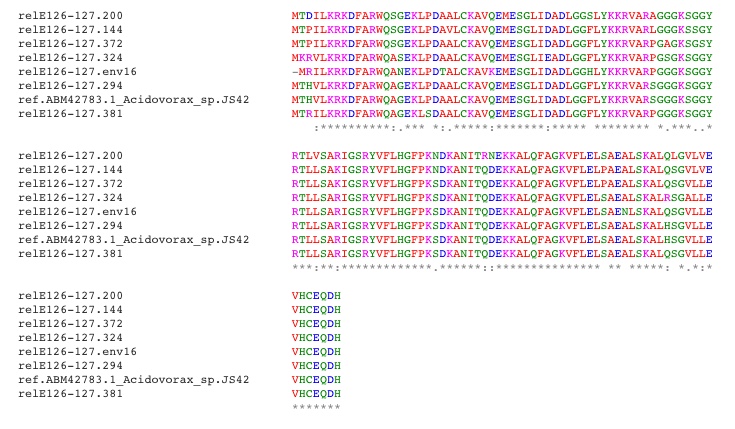


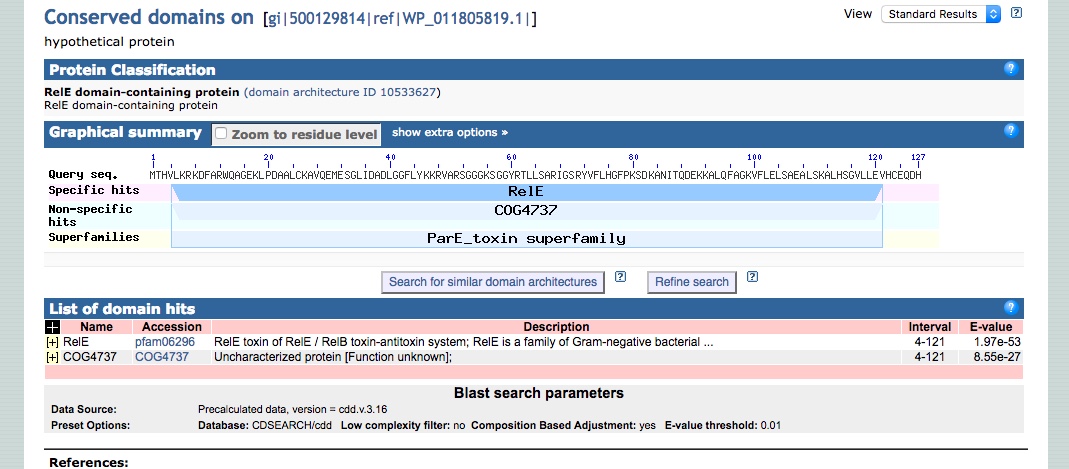


RHH/relE 78/111


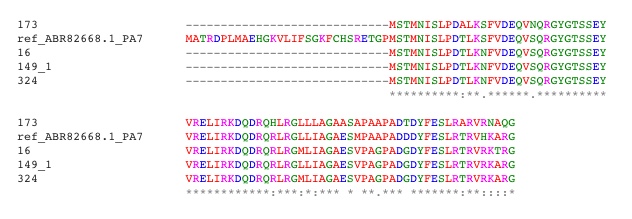


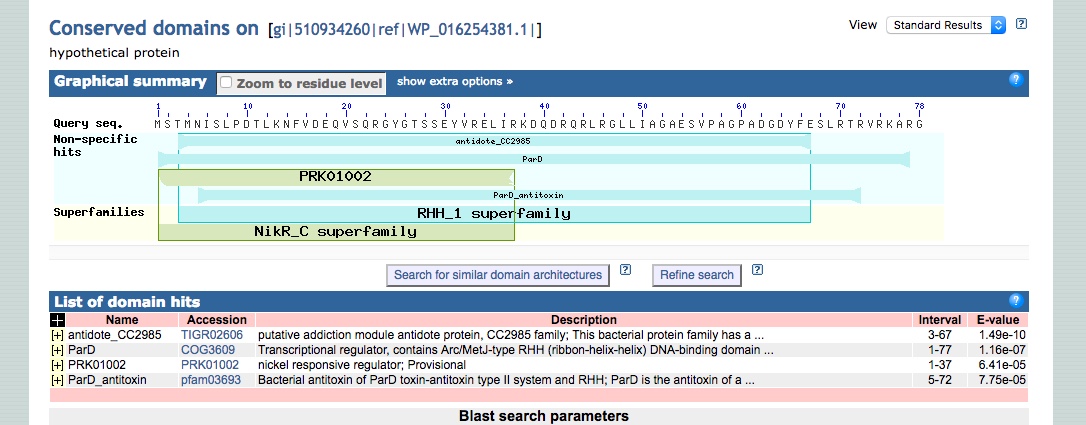


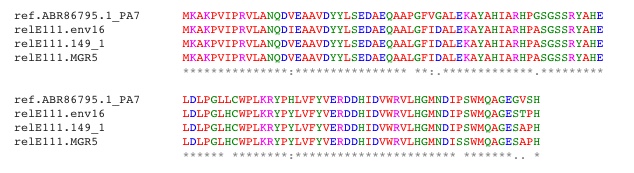


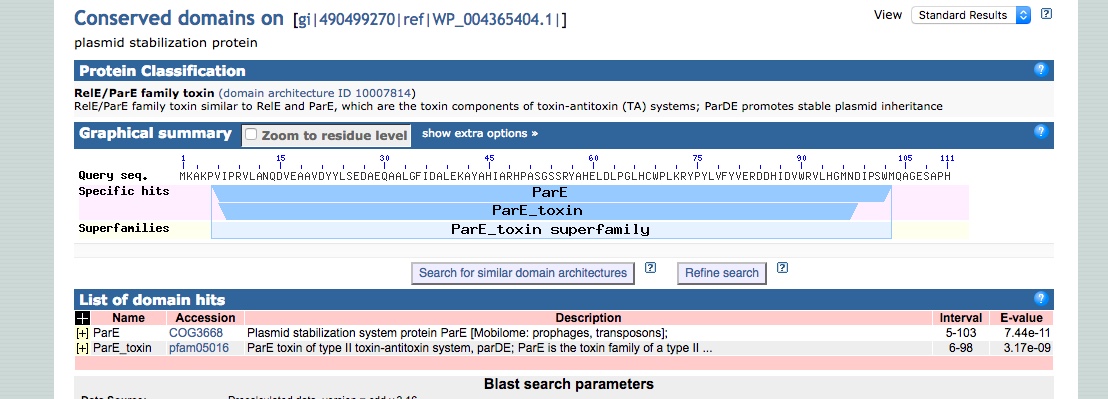


RHH/PIN 84/139


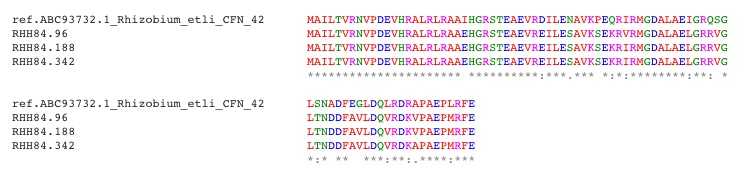


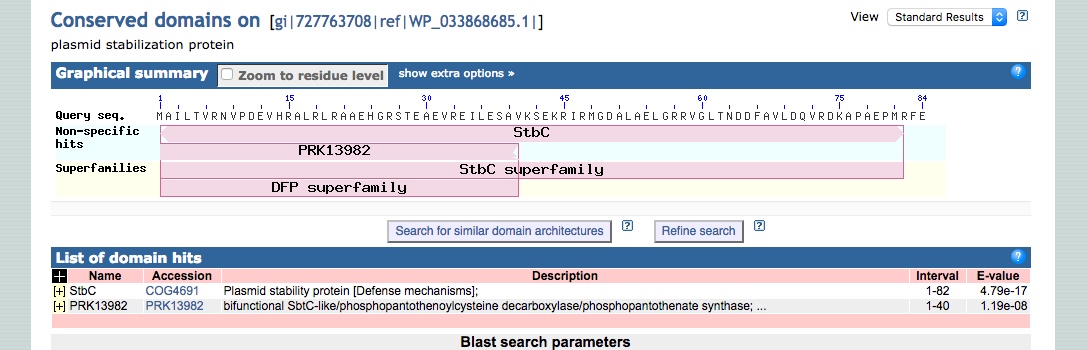


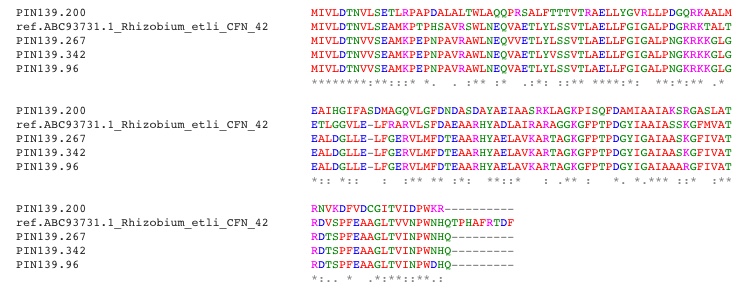


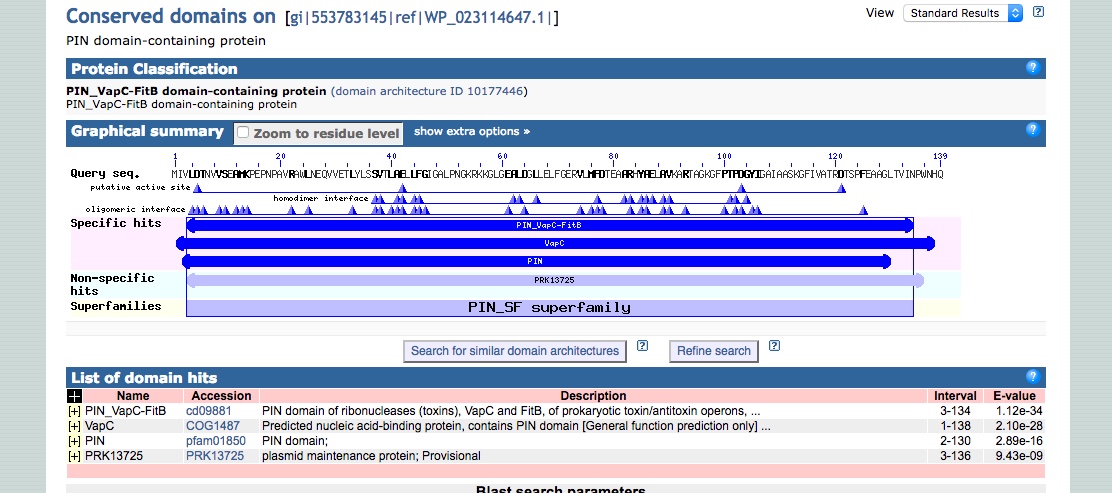


COG5606/relE 111/125


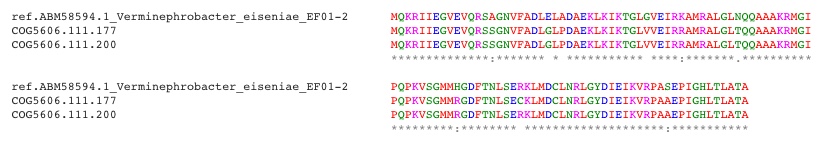


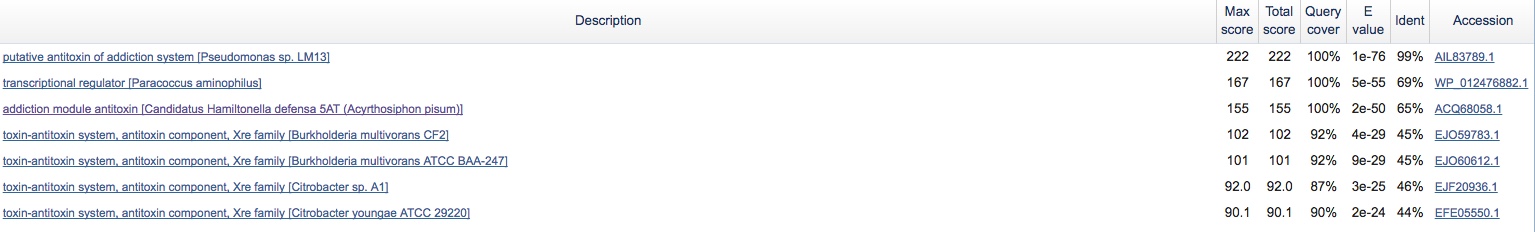


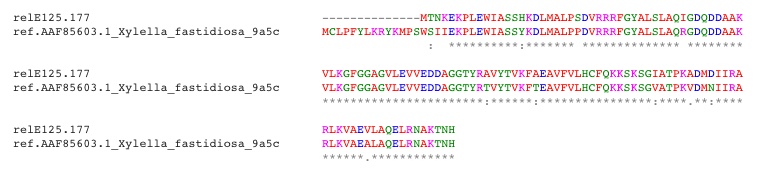


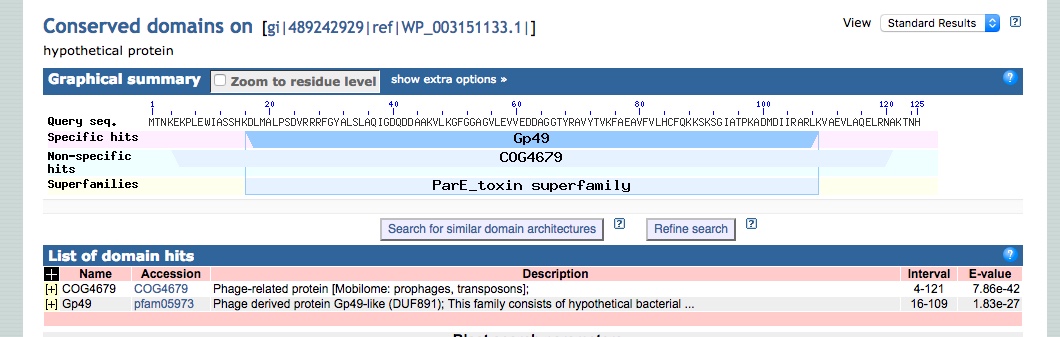


vapB/PIN 83/131


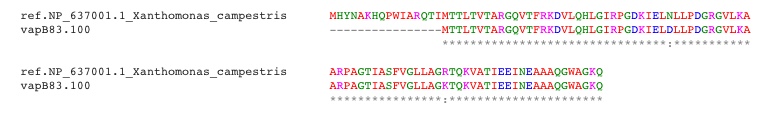


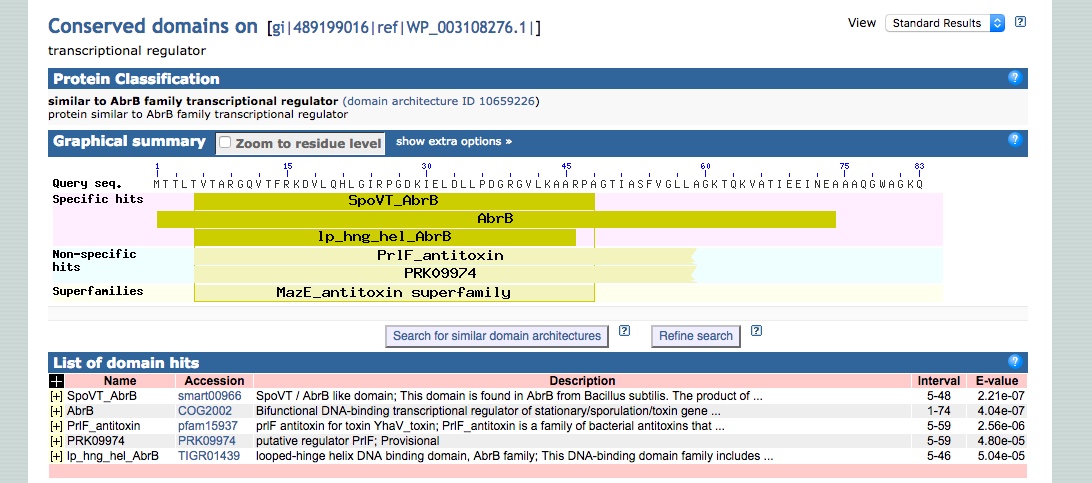


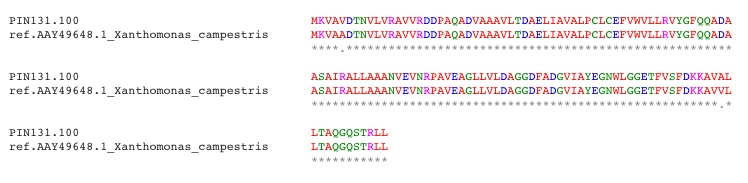


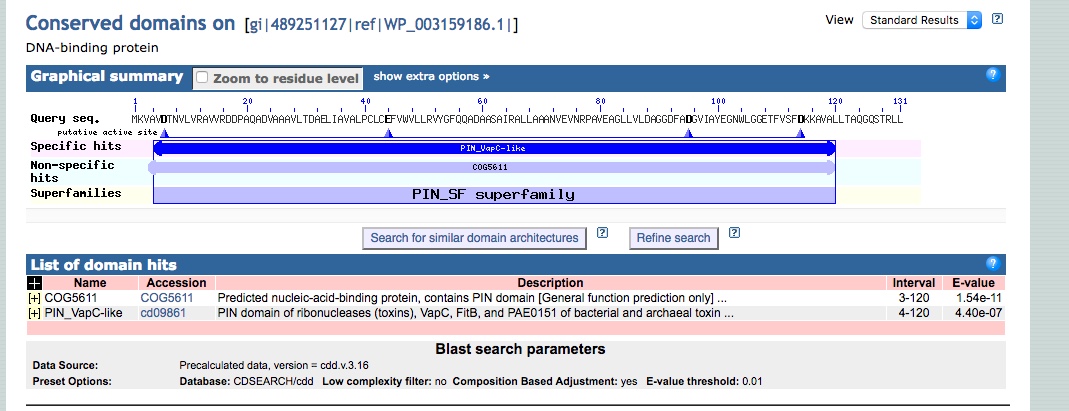


vapB/vapC 79/136


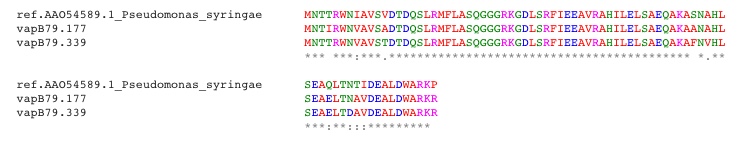


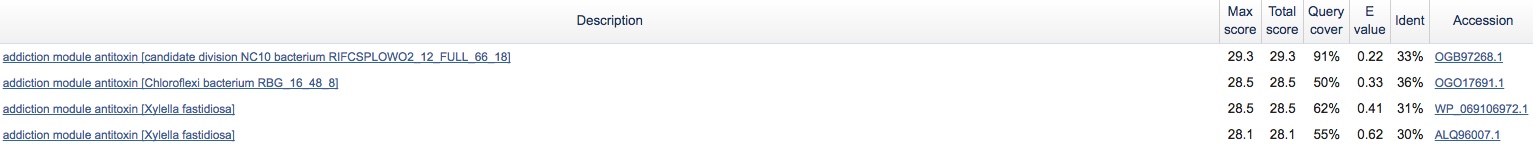


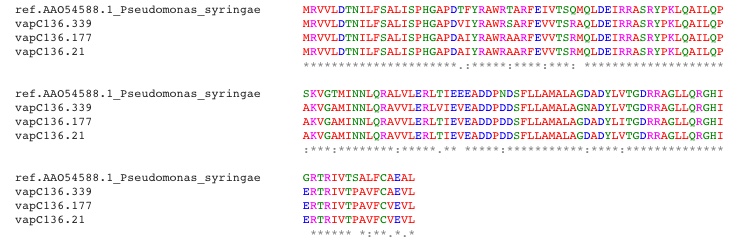


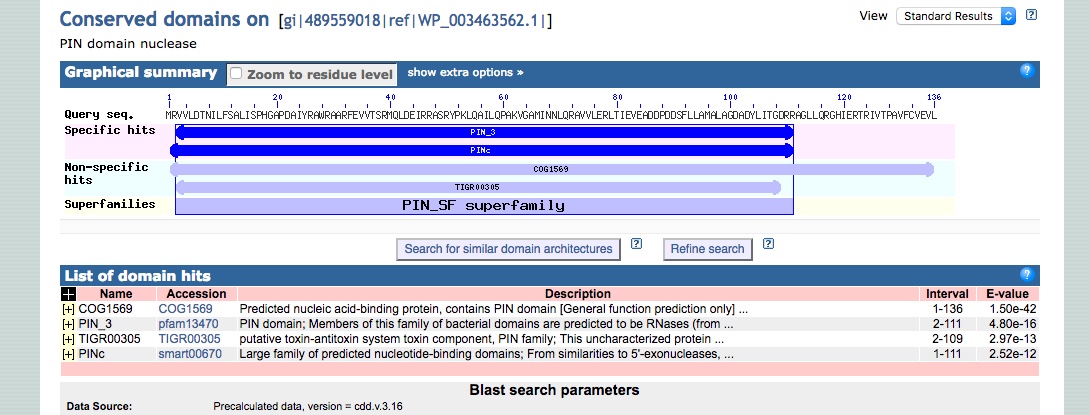


hicBA 160-231/59-60

No reference from TAfinder


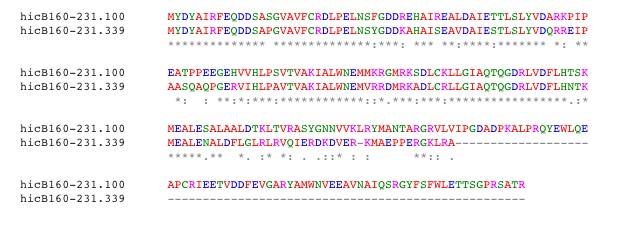


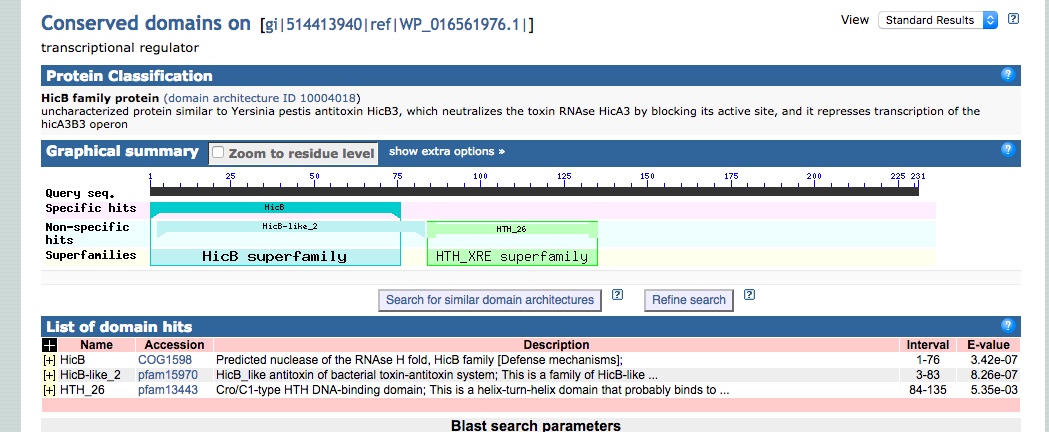


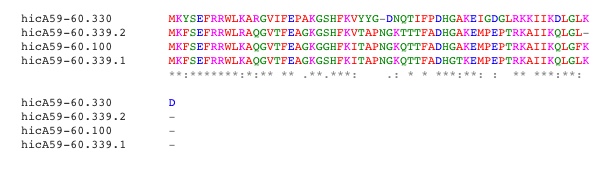


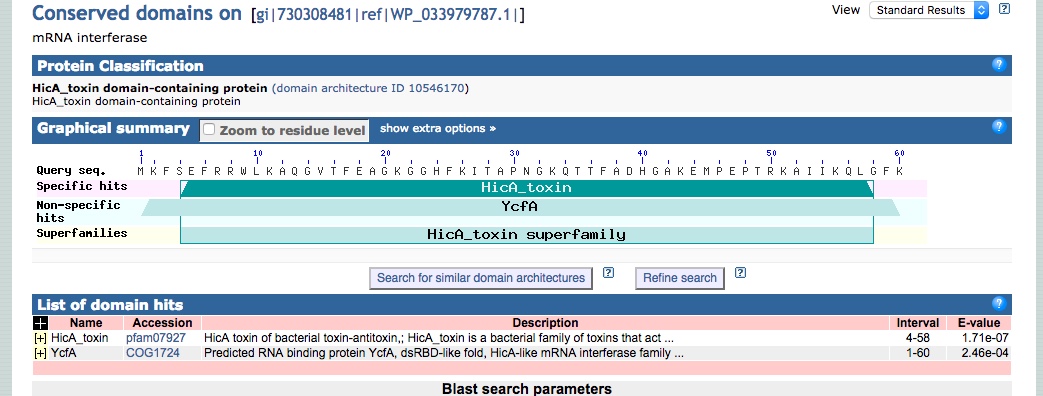


COG5642/COG5654 122-123/175 - unverified


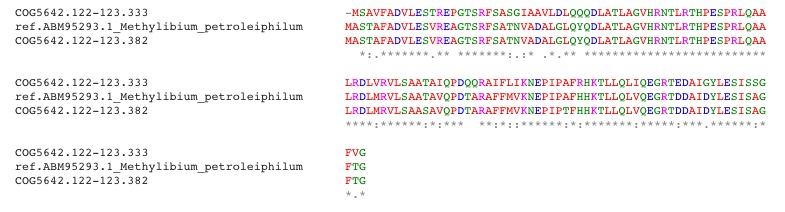


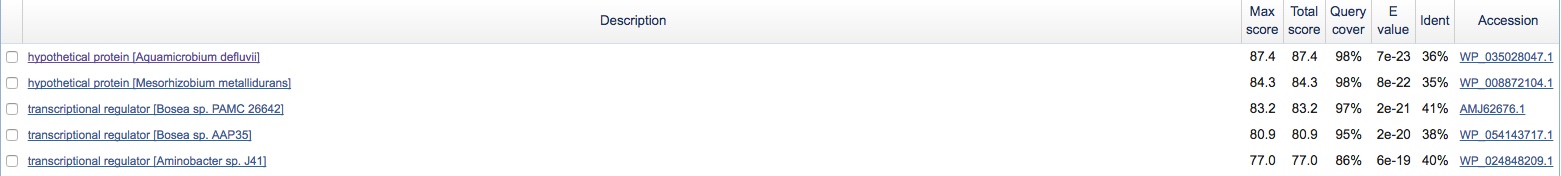


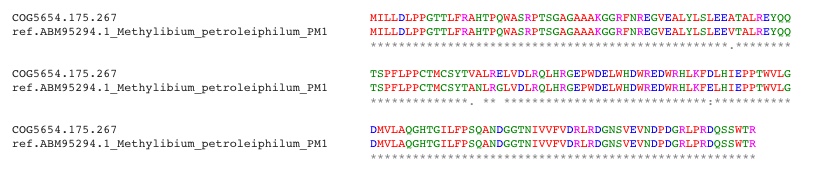


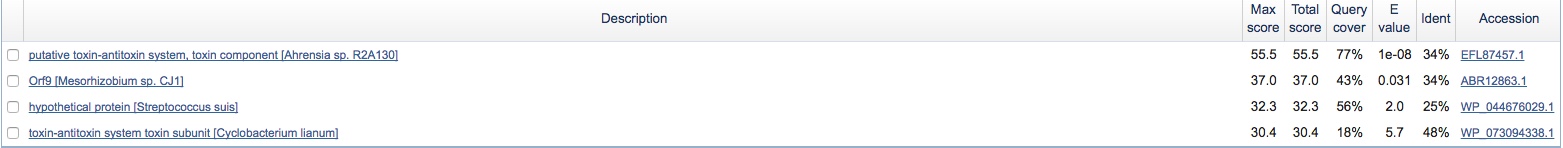


HEPN/MNT 128/96 - unverified


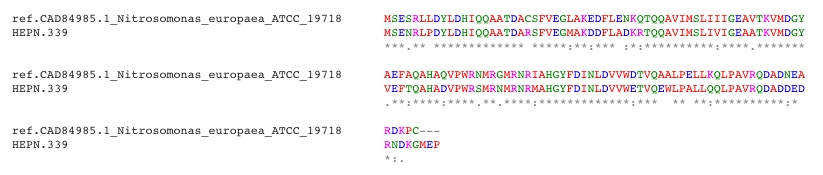


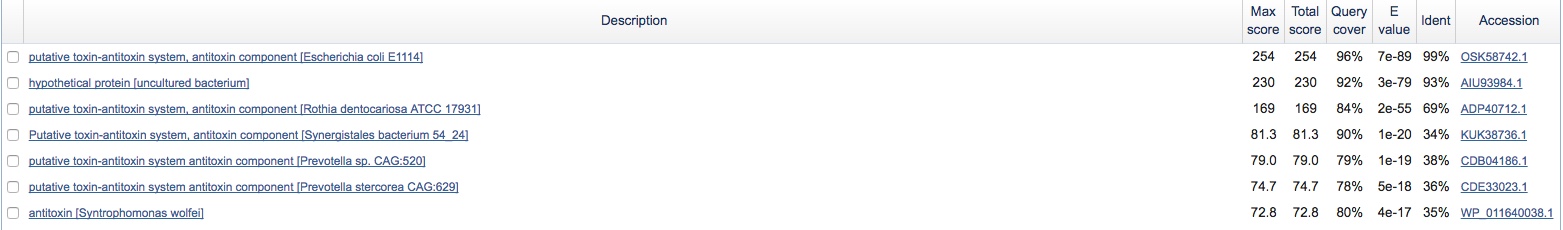


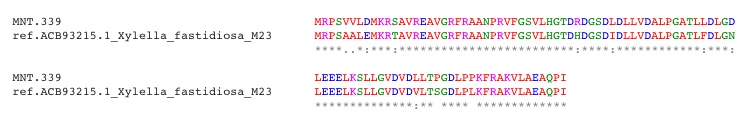


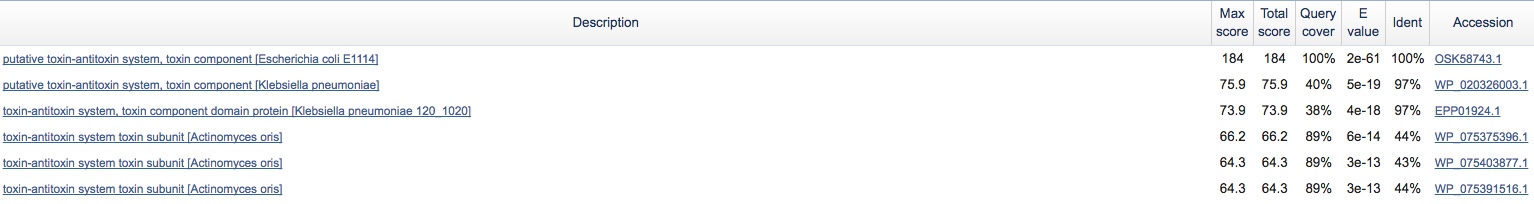


PHD/relE 83/84


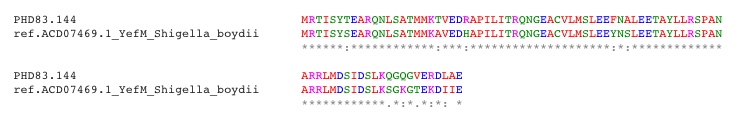


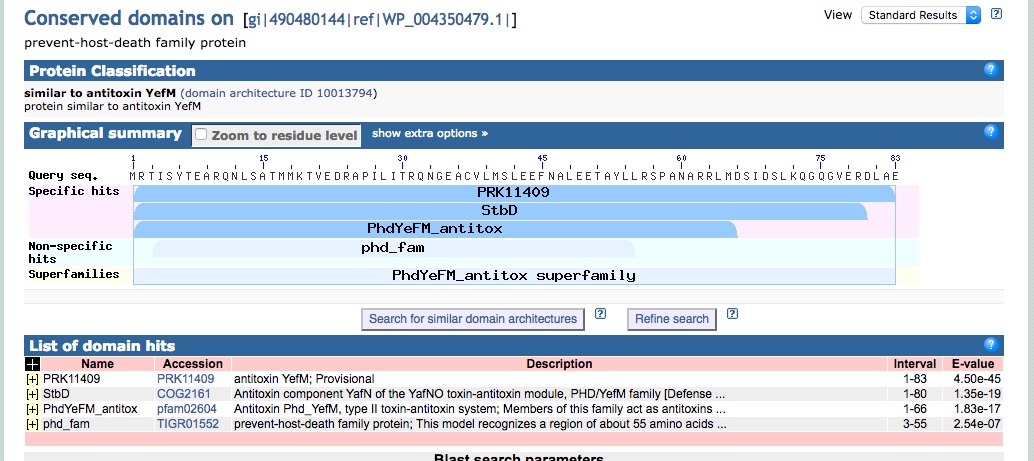


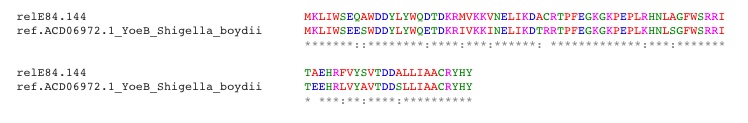


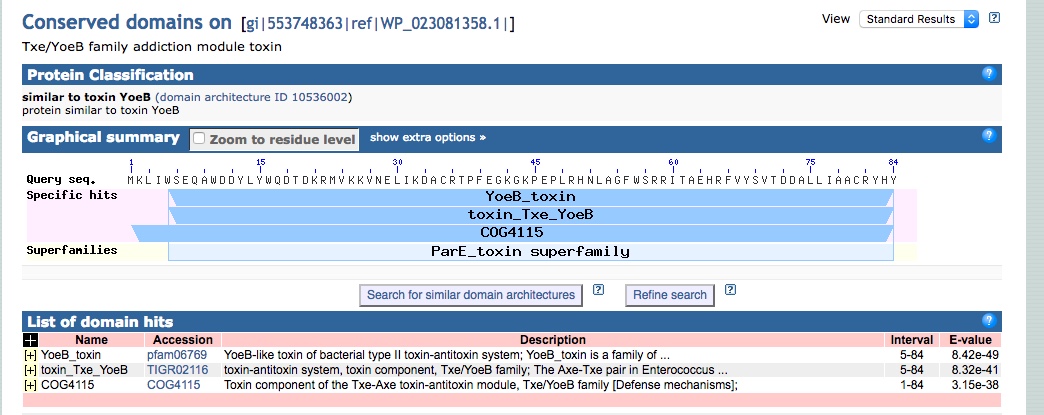


COG5606/relE 106/120-121


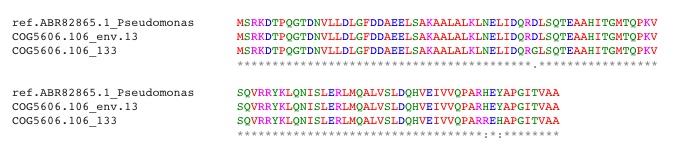


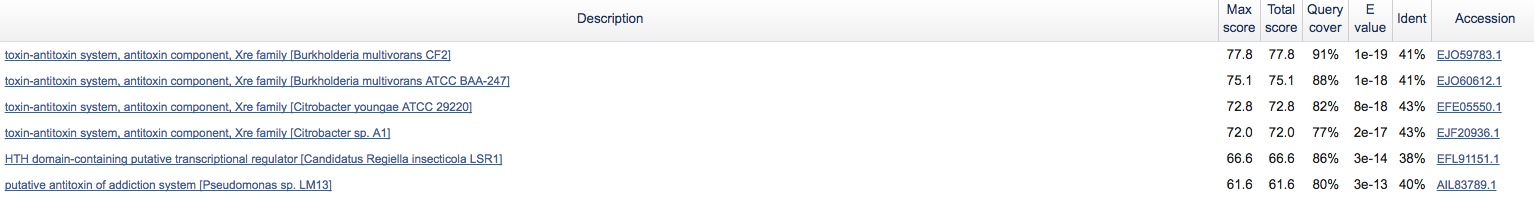


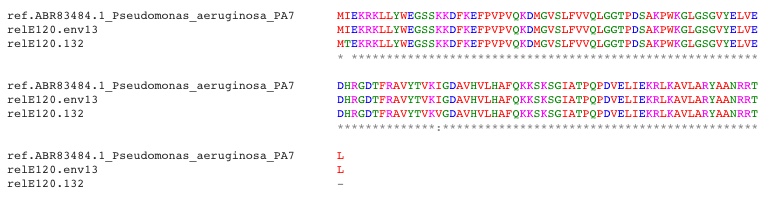


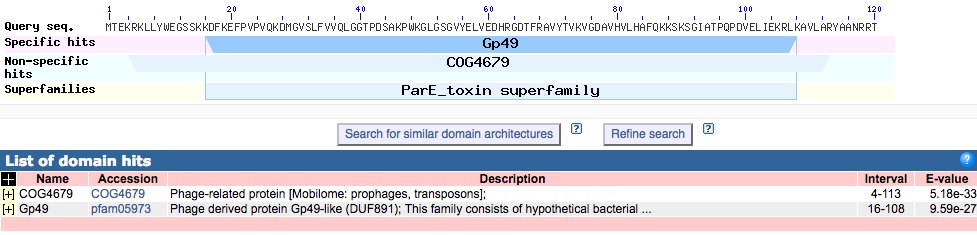


abrB/PIN 87/129


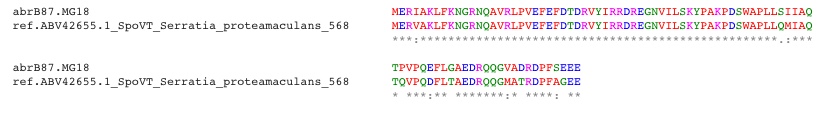


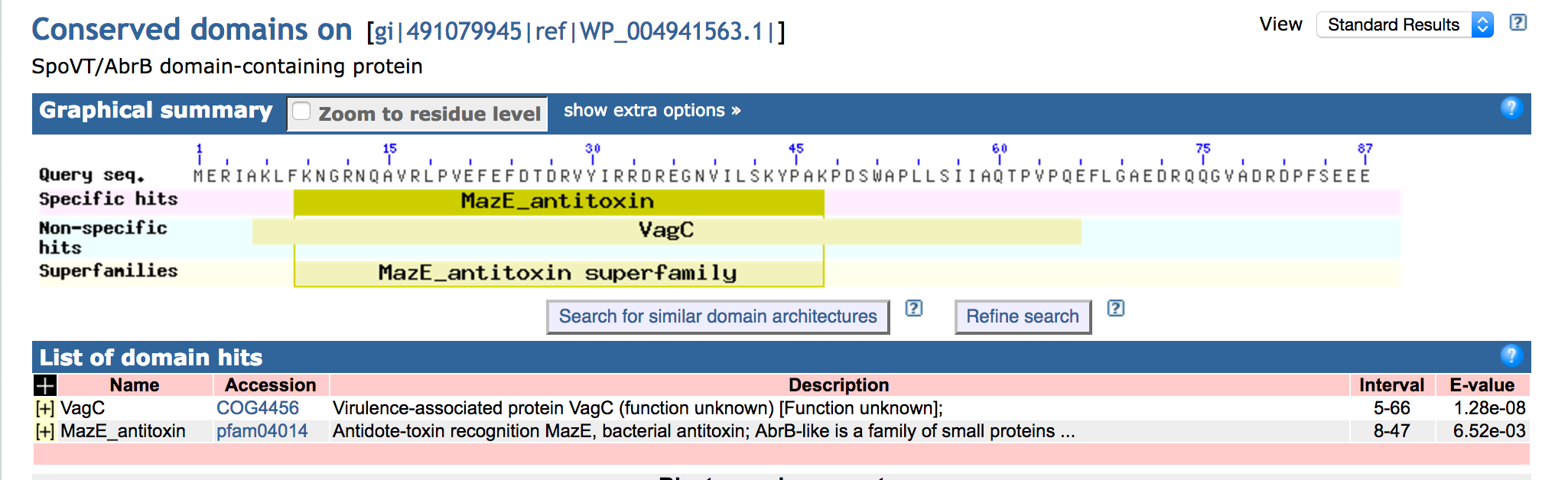


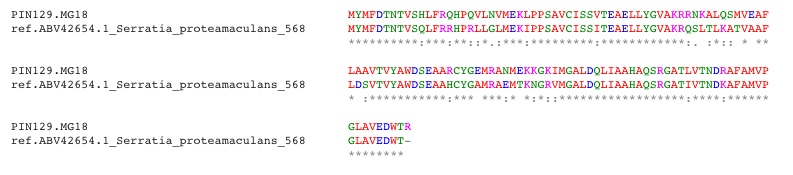


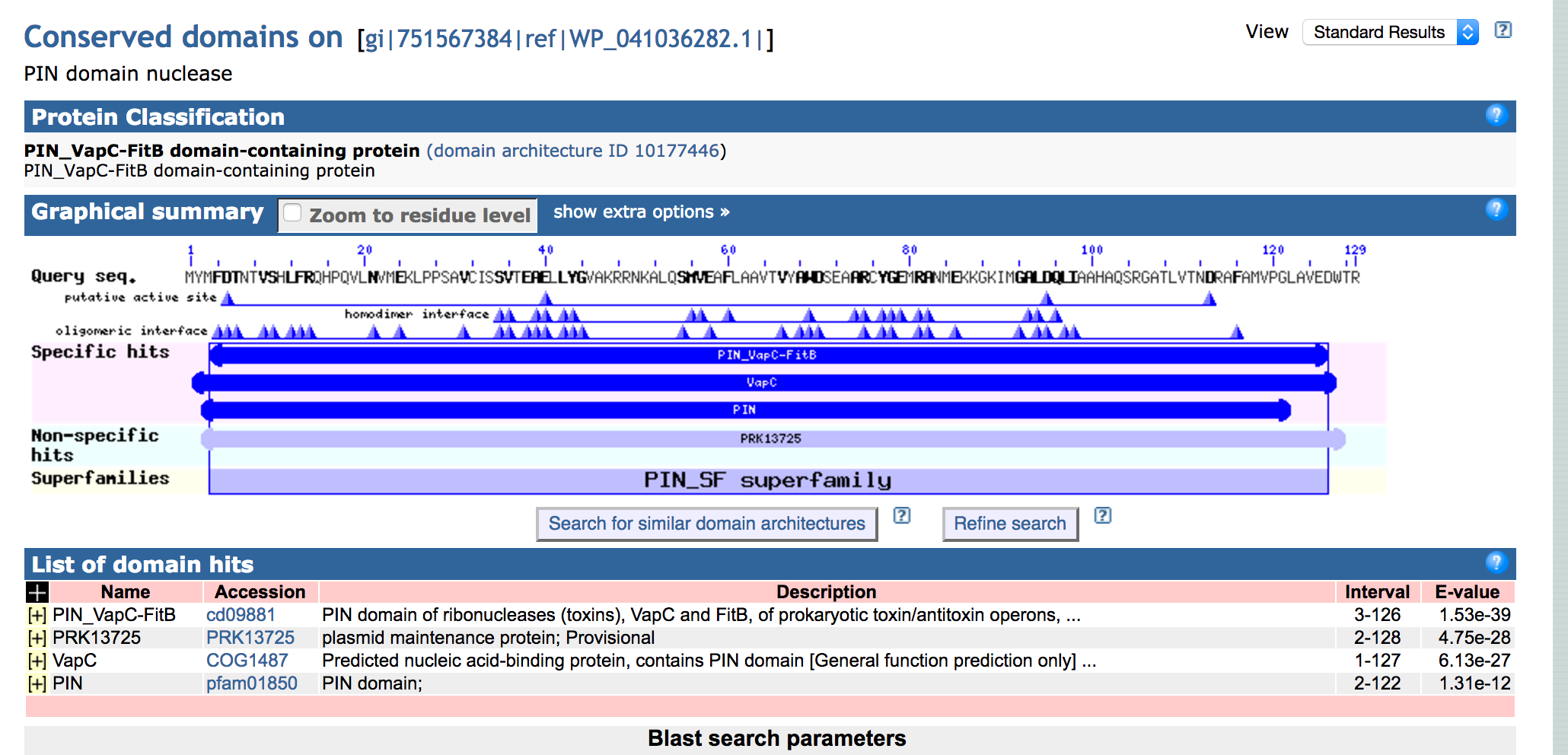


arsR/COG3832 115/117 - unverified


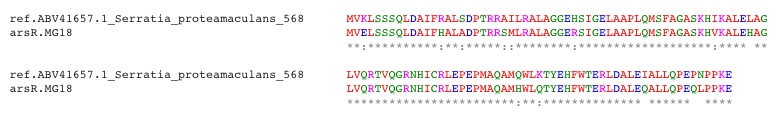


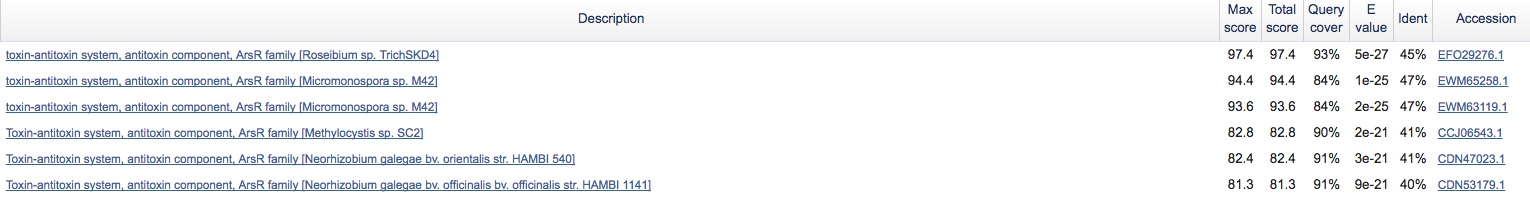


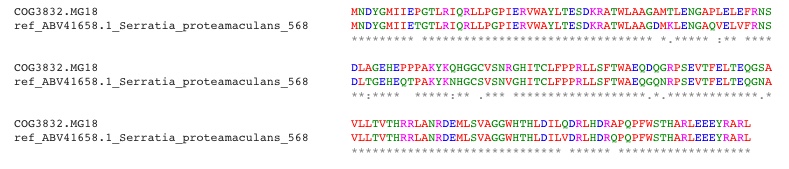


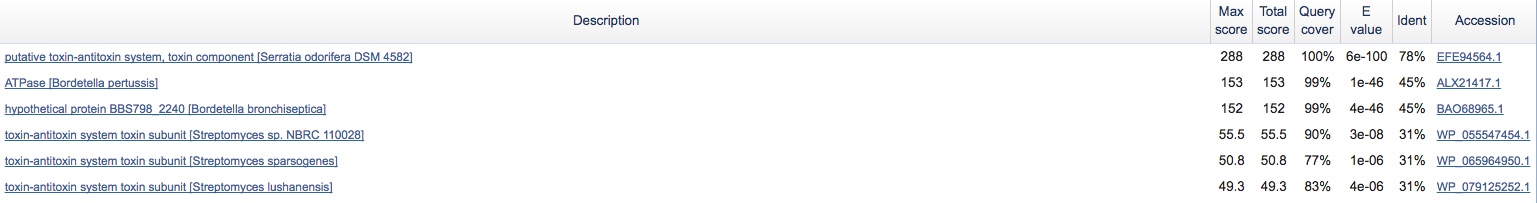


COG5606/relE 112/120


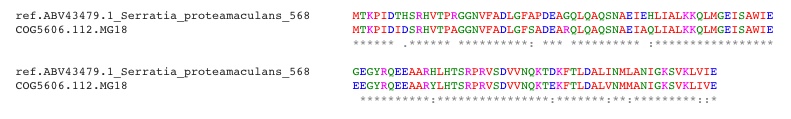


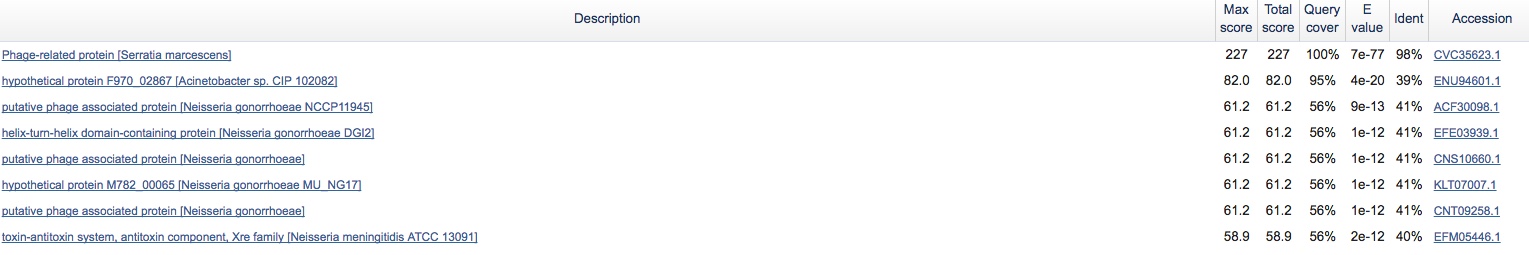


hicBA 121/91

PHD/PIN 77/128

relBE 90/97

RHH/PIN 73/126

RHH/relE 78/109

xre/hipA 103/429

xre/relE 100/117
